# Planar Group Functionalization of Quasi-Discrete Pores in Metal–Organic Frameworks for Enhanced Isomeric Separation in Simulated Moving Bed Processes

**DOI:** 10.1021/acscentsci.4c00876

**Published:** 2024-08-27

**Authors:** Zhe Chu, Jiaqi Li, Fuqiang Chen, Yifeng Cao, Lihang Chen, Feng Zhou, Huixia Ma, Qiwei Yang, Zhiguo Zhang, Kai Qiao, Qilong Ren, Zongbi Bao

**Affiliations:** † Key Laboratory of Biomass Chemical Engineering of ministry of Education, College of Chemical and Biological Engineering, Zhejiang University, 866 Yuhangtang Road, Hangzhou 310058, P. R. China; ‡ Institute of Zhejiang University-Quzhou, Quzhou 324000, P. R. China; § SINOPEC (Dalian) Research Institute of Petroleum and Petrochemicals Co., Ltd., 96 Nankai Street, Lvshunkou District, Dalian 116045, P.R. China

## Abstract

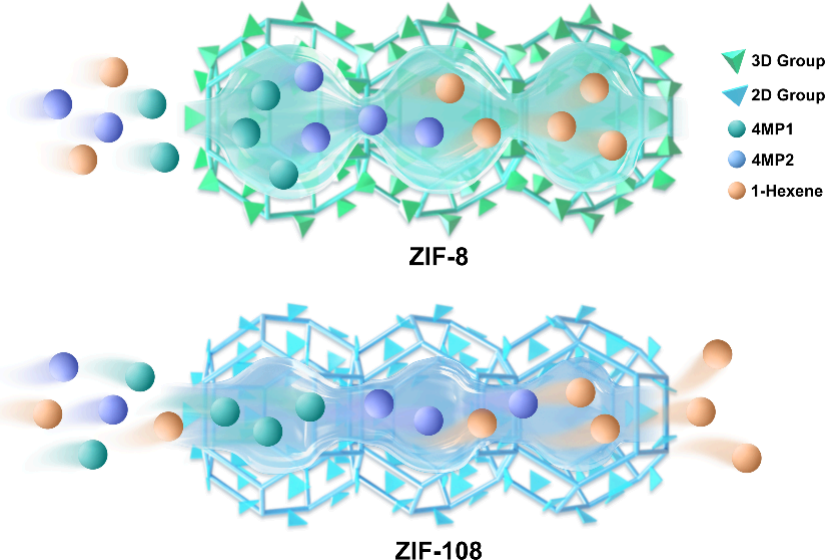

The efficient separation of 4-methyl-1-pentene (4MP1) from its structural isomers is crucial for industrial applications but remains challenging due to the similar physicochemical properties of these compounds. This study introduces a novel strategy using metal–organic frameworks (MOFs), specifically an engineered variant of ZIF-108, which demonstrates remarkable improvements in the thermodynamic and kinetic properties for 4MP1 separation. By substituting the methyl groups in ZIF-8 with planar nitro groups, we achieved a strategic resizing of the pore windows and cavity dimensions in ZIF-108. This adjustment not only enhanced the molecular affinity and selectivity toward 4MP1 but also facilitated a diffusion rate that is 164 times faster than that observed in ZIF-8. These properties significantly elevated the performance of ZIF-108 in simulated moving bed (SMB) processes, achieving up to 96.5% recovery of high-purity 4MP1, outperforming traditional adsorbents. Comprehensive characterization, including density functional theory (DFT) calculations and molecular dynamics (MD) simulations, provided insights into the interactions and the stability of the adsorption process. The findings suggest that the strategic modification of the pore architecture in MOFs holds significant potential for optimizing the separation processes of industrially relevant mixtures.

## Abstract 2

Simultaneous engineering of cavity and window within quasi-discrete pore structure in metal−organic frameworks enhances the thermodynamic and kinetic separation of 4-methyl-1-pentene from its isomers.

## Introduction

4-Methyl-1-pentene (4MP1), a notable alpha-olefin recognized for its exceptional properties, serves as a versatile monomer in the synthesis of poly(4-methyl-1-pentene) (PMP) and other innovative polymers.^1−5^ PMP, distinguished by its outstanding corrosion and heat resistance as well as superior electrical insulation properties, is a key material in various advanced applications. These include extracorporeal membrane oxygenation (EMCO) systems, also known as artificial lungs, solar cell components, flexible printed circuit substrates, and other cutting-edge materials.^6,7^ 4MP1 is predominantly produced through the dimerization of propylene, during which it is accompanied by low concentrations (approximately 5%) of 4-methyl-2-pentene (4MP2) and 1-Hexene (1-Hex).^8^ This mixture complicates its utility in various end-use scenarios due to the challenge of separating these olefin isomers. Currently, the separation process relies heavily on energy-intensive multistep distillation.^9^ Given that 4MP1 is the most thermodynamically unstable methylpentene isomer, distillation is not an optimal separation method. Therefore, the development of nonthermal separation technologies is highly desirable.

Simulated moving bed (SMB) chromatography has emerged as a prominent method for continuous adsorption separation, particularly successful in isolating pharmaceuticals and xylenes due to its high purity outputs and low energy requirements.^10−15^ Its ability to perform separations at ambient temperatures makes it especially suitable for thermally labile compounds such as 4MP1, thereby offering extensive potential for various applications. The success of SMB hinges on the performance of the adsorbent, which must exhibit exceptional chromatographic separation capabilities and rapid mass transfer to sustain continuous adsorption and elution cycles. However, zeolites, commonly employed in SMB processes, often encounter limitations such as restricted mass transfer rates and high regeneration demands.^16^ This highlights the urgent need for the development of more efficient adsorbents that meet stringent kinetic and thermodynamic criteria.

Metal–organic frameworks (MOFs), also known as porous coordination polymers, are garnering increasing attention in adsorption separation due to their tailorable pore structure and customizable functionalities.^17−36^ Recent advancements have enabled precise tuning of the pore structures of MOFs, allowing for the regulation of the adsorption and diffusion rates of guest molecules.^37−39^ This ability to adjust adsorption kinetics makes MOFs highly promising for use in simulated moving bed (SMB) processes. Nevertheless, controlling the adsorption kinetics of guest molecules is often achieved by substituting metal ions or adjusting ligand lengths to regulate the pore size of MOFs.^40−42^ While increasing the pore size enhances diffusion, it simultaneously reduces the micropore confinement effect, thereby diminishing the affinity for guest molecules. Overcoming this dilemma and achieving a balance between enhanced diffusion kinetics and strong affinity for guest molecules remain substantial challenges, critical for optimizing SMB applications.

**Scheme 1:**
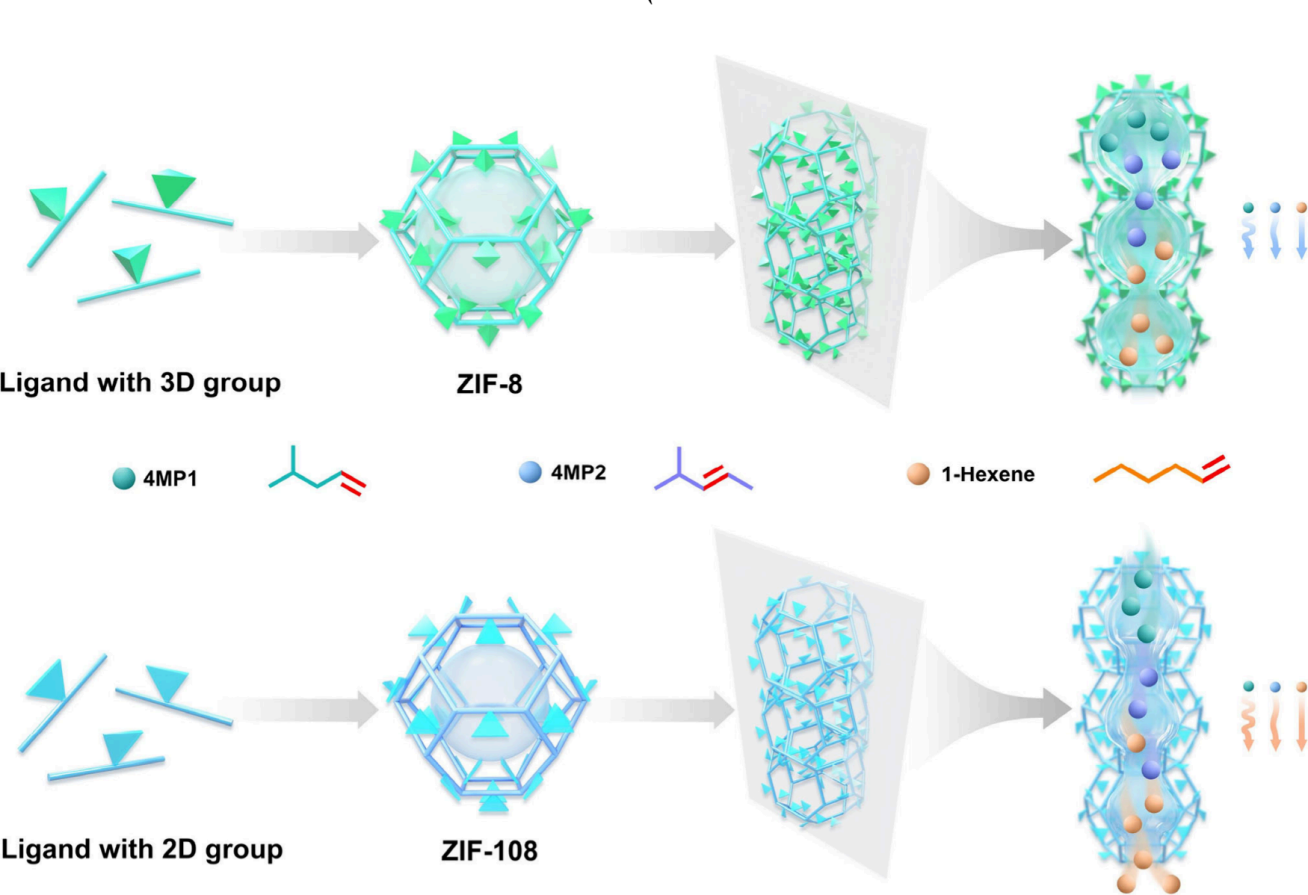
Schematic Illustration of Simultaneous Engineering of Cavity and Window within Quasi-Discrete Pore Structure in MOFs for Enhancing Thermodynamic-Kinetic Separation of 4MP1 from its Isomers

In this work, ZIF-8 with a classical SOD topology was used because the large cage of 11.2 Å in ZIF-8 offers sufficient space to accommodate long olefin molecules, while the narrow windows (3.3 Å) potentially improve the kinetic separation performance. However, the window size closely matches the minimum cross section of these molecules (Figure S1), possibly creating a significant diffusion barrier that could reduce the efficiency of the SMB process. Given that the functional groups of the ligands are uniformly distributed around the cavity and pore windows, modifying these groups will affect both the pore cavity and the pore window sizes. It remains challenging to increase the pore window size to enhance guest molecule diffusion without enlarging the pore cavity and weakening the micropore confinement effect. To address this issue, we ingeniously replaced the three-dimensional methyl functional groups on the ligands with two-dimensional planar nitro functional groups, resulting in isostructural ZIF-108. The elongated nitro groups reduce the radial size of the pore cavity, enhancing the micropore confinement effects of guest molecules, while the thinner dimension of the nitro groups increases the pore window size, facilitating the diffusion of guest molecules (Scheme 1). As anticipated, the diffusion rates and affinity of components in ZIF-108 are significantly improved compared with those in ZIF-8. Specifically, the diffusion rate of 4MP1 in ZIF-108 is 164 times greater than that in ZIF-8. Additionally, at 303 K and 27 kPa, the uptake ratios of 4MP2/4MP1 and 1-Hex/4MP1 are 1.80 and 1.64, respectively, markedly exceeding those of other MOFs and zeolites. High Henry’s selectivities for 4MP2/4MP1 (4.13) and 1-Hex/4MP1 (10.74), derived from liquid-phase adsorption isotherms obtained through frontier chromatography, underscore the high feasibility of ZIF-108 for the SMB process. Remarkably, SMB simulations show that ZIF-108 can achieve high-purity 4MP1 with a recovery rate of up to 96.5% from 4MP1/4MP2/1-Hex mixtures, demonstrating its considerable potential for industrial SMB separation processes. Density functional theory (DFT) calculations and molecular dynamics (MD) simulations highlight the crucial role of multidimensional regulation of functional groups on ligands in simultaneously engineering pore window and cavity sizes to enhance thermodynamic and kinetic separation performance.

## Results and Discussion

**Figure 1:**
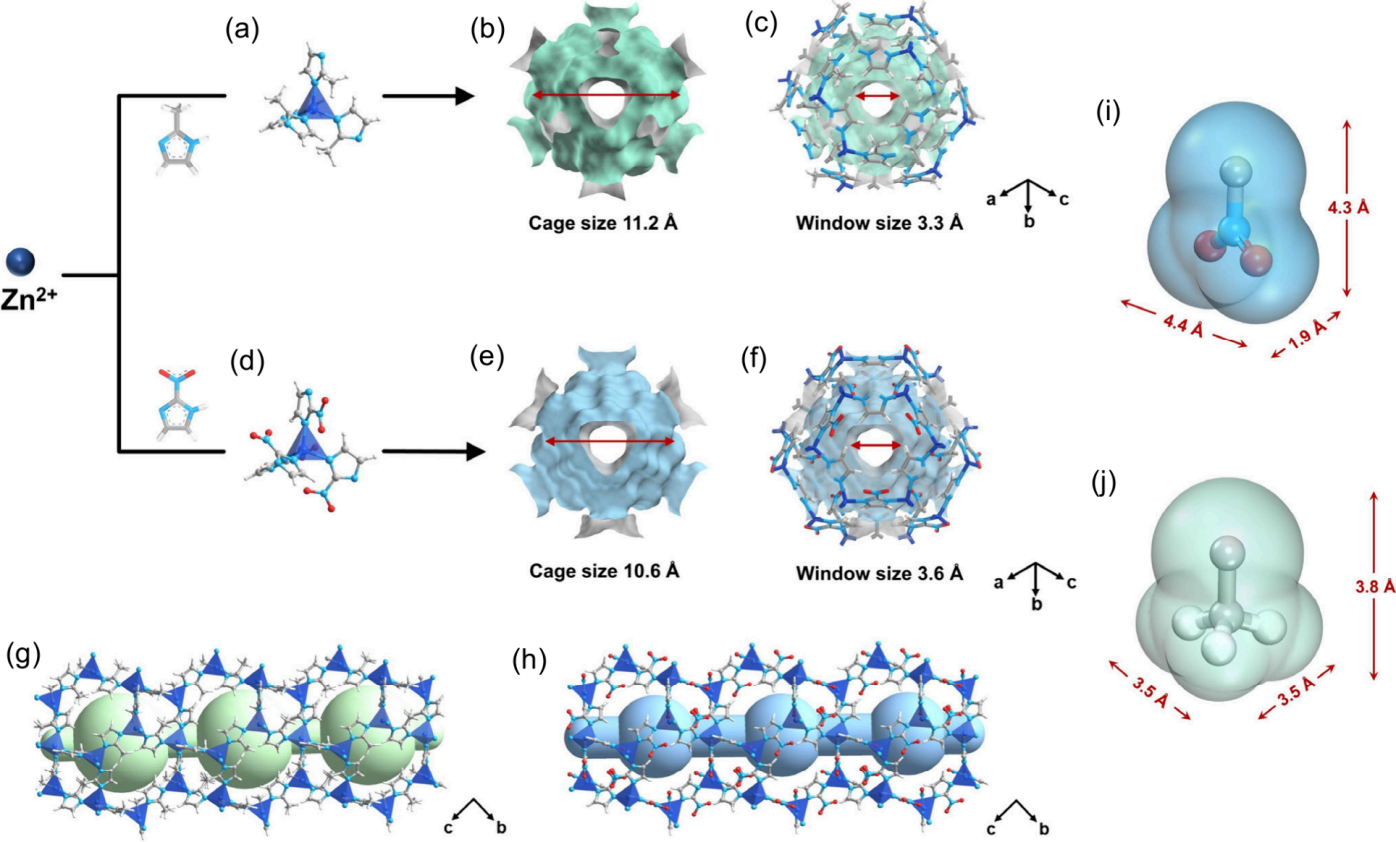
Schematic representation of the synthesis of ZIF-8 and ZIF-108 and their corresponding structures. The SBUs structural unit of ZIF-8 (a) and ZIF-108 (d); the cage size of ZIF-8 (b) and ZIF-108 (e); the window size of ZIF-8 (c) and ZIF-108 (f). Sharing of the hexagonal faces with neighboring cages leads to 1D channels of ZIF-8 (g) and ZIF-108 (h). The dimensional sizes of (i) methyl group and (j) nitro group.

ZIF-8 with high crystallinity was successfully synthesized following previously reported methods,^43^ as verified by single-crystal X-ray diffraction (SCXRD) data (Table S1). The Zn(II) ion is tetrahedrally coordinated by four nitrogen atoms from 2-methylimidazole, forming the Zn(mim)_4_ secondary building units (SBU) (Figure 1a), which further construct large truncated octahedra with a cage size of 11.2 Å. These octahedra feature six square faces and eight hexagonal faces that are shared with adjacent cages (Figure 1b). This arrangement results in 1D channels along the body diagonals of the cubic lattice, as depicted in Figure 1g. Additionally, the three-dimensional methyl group is oriented toward the pore window, restricting the pore window size to 3.3 Å (Figure 1c), which may enable regulation of the gas diffusion behavior. Similarly, ZIF-108, with the same topology as ZIF-8, was fabricated by replacing three-dimensional methyl groups from 2-methylimidazole with two-dimensional nitro groups (Figure 1d). The plane formed by the 2D nitro group in ZIF-108 is tangent to the 1D pore channel; thus, the elongated nitro groups reduce the radial size of the pore cavity to 10.6 Å (Figure 1e), further enhancing the micropore confinement effects of guest molecules. Meanwhile, the thinner dimension of nitro groups increases the pore window size by up to 3.6 Å (Figure 1f), facilitating the diffusion of guest molecules. The quasipore structure, characterized by a reduced pore cavity size and enlarged pore windows (Figure 1h), is expected to simultaneously enhance the thermodynamic affinity and increase the diffusion rates of guest molecules. X-ray photoelectron spectroscopy (XPS) was performed to investigate the chemical compositions of ZIF-8 and ZIF-108 (Figure S2). ZIF-8 and ZIF-108 possess similar chemical compositions (Figure S2a). The high-resolution N 1s spectra indicate that, in comparison to ZIF-8, ZIF-108 exhibits additional characteristic peaks at 405.1 eV (C–N) and 406.0 eV (N–O) (Figure S2c), thereby confirming the presence of nitro functional groups in ZIF-108. Fourier-transform infrared (FT-IR) spectra was further conducted to investigate the differences in the chemical compositions of ZIF-8 and ZIF-108 (Figure S3). Both ZIF-8 and ZIF-108 possess characteristic peaks of the stretching of imidazole rings (1568–1650 cm^–1^). Additionally, the presence of asymmetric and symmetric stretching peaks of −NO_2_ (1527 and 1350 cm^–1^) in ZIF-108 confirms the presence of nitro functional groups. To observe the morphology of these materials, scanning electron microscopy (SEM) were performed. As displayed in Figure S4 and Figure S5, both ZIF-8 and ZIF-108 exhibit stacked block-like morphologies, and the uniform particle sizes make them suitable as packing materials for chromatography and adsorption beds. Upon activation, the morphology and size of the materials remain nearly unchanged, indicating their excellent durability. Additionally, EDS mapping results reveal the uniform distribution of elements within the materials (Figure S4c–g and Figure S5c–g).

**Figure 2:**
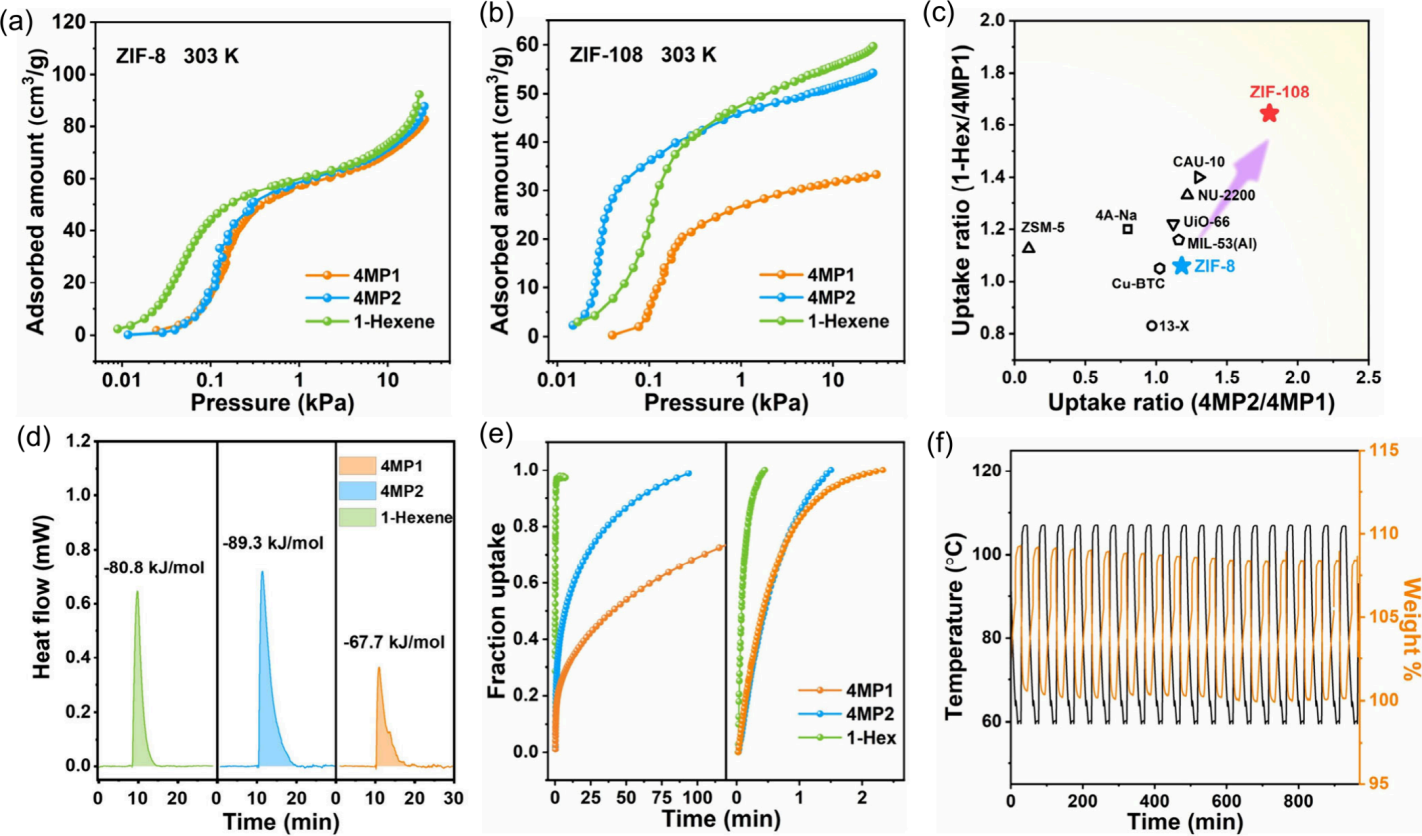
Adsorption isotherms of 4MP1, 4MP2, and 1-Hexneon ZIF-8 (a) and ZIF-108 (b) at 303 K. Comparison of the uptake ratios of 4MP2/4MP1 and 1-Hexene/4MP1 among various materials (c). (d) The adsorption heats of 4MP1, 4MP2, and 1-Hexne on ZIF-108 determined by DSC. (e) Time-dependent vapor uptake profiles of 4MP1, 4MP2, and 1-Hexne at 303 K and 0.45 kPa on ZIF-8 and ZIF-108. (f) The cycling adsorption–desorption tests of 1-Hexene on ZIF-108 for 20 consecutive cycles on TGA instrument at 303 K.

The porous structure of ZIF-8 and ZIF-108 was further verified by N_2_ adsorption tests at 77 K (Figure S6 and Table S2). The Brunauer–Emmett–Teller (BET) specific surface areas of ZIF-8 and ZIF-108 were calculated to be 1752.3 and 956.4 m^2^/g, respectively, and the pore volumes were 0.67 and 0.62 cm^3^/g, respectively. The pore size distributions of ZIF-8 and ZIF-108 were calculated by the Horvath—Kawazoe model (Figure S7). The cavity size of ZIF-8 is slightly higher than that of ZIF-108, consistent with the results of SCXRD analysis. Inspired by the well-developed porosity of these MOFs, adsorption isotherms of 4MP1, 4MP2, and 1-Hex at different temperatures were collected (Figure 2a,b and Figures S8 and S9). ZIF-8 demonstrated a high adsorption uptake of these olefin isomers due to the high BET specific surface areas and pore volumes. The uptakes of 4MP1, 4MP2, and 1-Hex on ZIF-8 at 303 K and 27 kPa reached 82.6, 87.6, and 92.3 cm^3^/g, respectively, resulting in low uptake ratios. During the low-pressure range, the uptakes of ZIF-8 follow the trend of 1-Hex > 4MP2 > 4MP1. This is because the pore cavity in ZIF-8 provides the strongest micropore confinement effect for the longest 1-Hex, while ZIF-8 demonstrates the weakest affinity for the shortest 4MP1, suggesting that ZIF-8 could effectively separate these components based on affinity discrepancies. By contrast, despite lower uptakes in ZIF-108 compared to ZIF-8, ZIF-108 displays a notably steeper isotherm for 4MP2, a slightly steeper isotherm for 1-Hexene, and a nearly flat adsorption isotherm for 4MP1, particularly at low pressures, indicating the enhanced affinity difference between 4MP1 and impurity (4MP2 and 1-Hexene) on ZIF-108. Due to the enhanced affinity difference between target component and impurities in ZIF-108, the uptake ratios of 4MP2/4MP1 and 1-Hex/4MP1 were significantly increased to 1.80 and 1.64, respectively, outperforming other reported MOFs and zeolites (Figure 2c), such as CAU-10 (1.41 and 1.31), UiO-66 (1.22 and 1.12), Cu-BTC (1.05 and 1.02), 13X zeolite (0.83 and 0.97), and ZSM-5 zeolite (1.13 and 0.10) (Table S3). Thermogravimetry-differential scanning calorimetry (TG-DSC) tests were conducted to determine the adsorption heats of these components on ZIF-8 and ZIF-108 (Figure 2d, Figure S9). As shown in Figure 2d, the adsorption heats of 1-Hex, 4MP2, and 4MP1 were evaluated to be 80.8, 89.3, and 67.7 kJ/mol, respectively, significantly higher than those recorded for ZIF-8 (Figure S10), consistent with the observed affinities for these components.

Considering the narrow pore windows in ZIF-8 and ZIF-108, which may impose varying diffusion restrictions on these components, we conducted further investigations into the kinetic adsorption performance of these components. Time-dependent gas uptake profiles of 1-Hex, 4MP2, and 4MP1 on ZIF-8 and ZIF-108 at 303 K and 0.45 kPa were collected. As illustrated in Figure 2e, 1-Hex, with the smallest cross-sectional size, reached adsorption equilibrium on ZIF-8 in merely 7 min. In contrast, 4MP2 and 4MP1, with larger cross-sectional size, required substantially longer times of 94 and 190 min, respectively, to reach equilibrium. The diffusion time constants were calculated using the classical micropore diffusion model^44^ to quantitatively compare the diffusion rates (Table S4). The diffusion time constants for 1-Hex, 4MP2, and 4MP1 on ZIF-8 were determined to be 5.16 × 10^–3^, 3.63 × 10^–5^, and 1.42 × 10^–5^ s^–1^, resulting in high kinetic selectivity for 1-Hex/4MP1 (363.6) and moderate kinetic selectivity for 4MP2/4MP1 (4.8). Despite the high kinetic selectivity for 4MP1 achieved on ZIF-8, the overall diffusion rates of these components are slow, impairing the continuous adsorption and desorption processes in SMB technologies. As anticipated, considerably faster diffusion rates of 1-Hex, 4MP1, and 4MP2 were achieved on ZIF-108, which has a slightly larger pore window. Specifically, the diffusion time constants for 4MP2 and 4MP1 increased by factors of 27.7 and 142.0, respectively, further demonstrating the critical role of quasi-discrete pore structure engineering in tuning diffusion behavior.

The substantial potential of ZIF-108 in the SMB processes prompted us to explore its stability. Powder X-ray diffraction measurements confirmed that the crystallinity of ZIF-8 and ZIF-108 remains intact after being exposed to air for 45 days (Figure S11). Additionally, N_2_ adsorption isotherms at 77 K demonstrated their maintained porosity after air exposure (Figure S4 and Table S2), indicating remarkable stability in air. The thermal stabilities of ZIF-8 and ZIF-108 were examined by thermogravimetric analysis (TGA). ZIF-8 does not experience structural collapse until 400 °C under an N_2_ atmosphere and exhibited robust thermal stability up to 350 °C (Figure S12). Motivated by the exceptional stability of ZIF-108, we conducted 20 consecutive cycles of adsorption–desorption tests of 1-Hex using a TGA instrument to investigate its regeneration ability. As seen from Figure 2f, the adsorption capacity of 1-Hex on ZIF-108 can be well maintained even after 20 cycles, underscoring its excellent regeneration performance.

**Figure 3:**
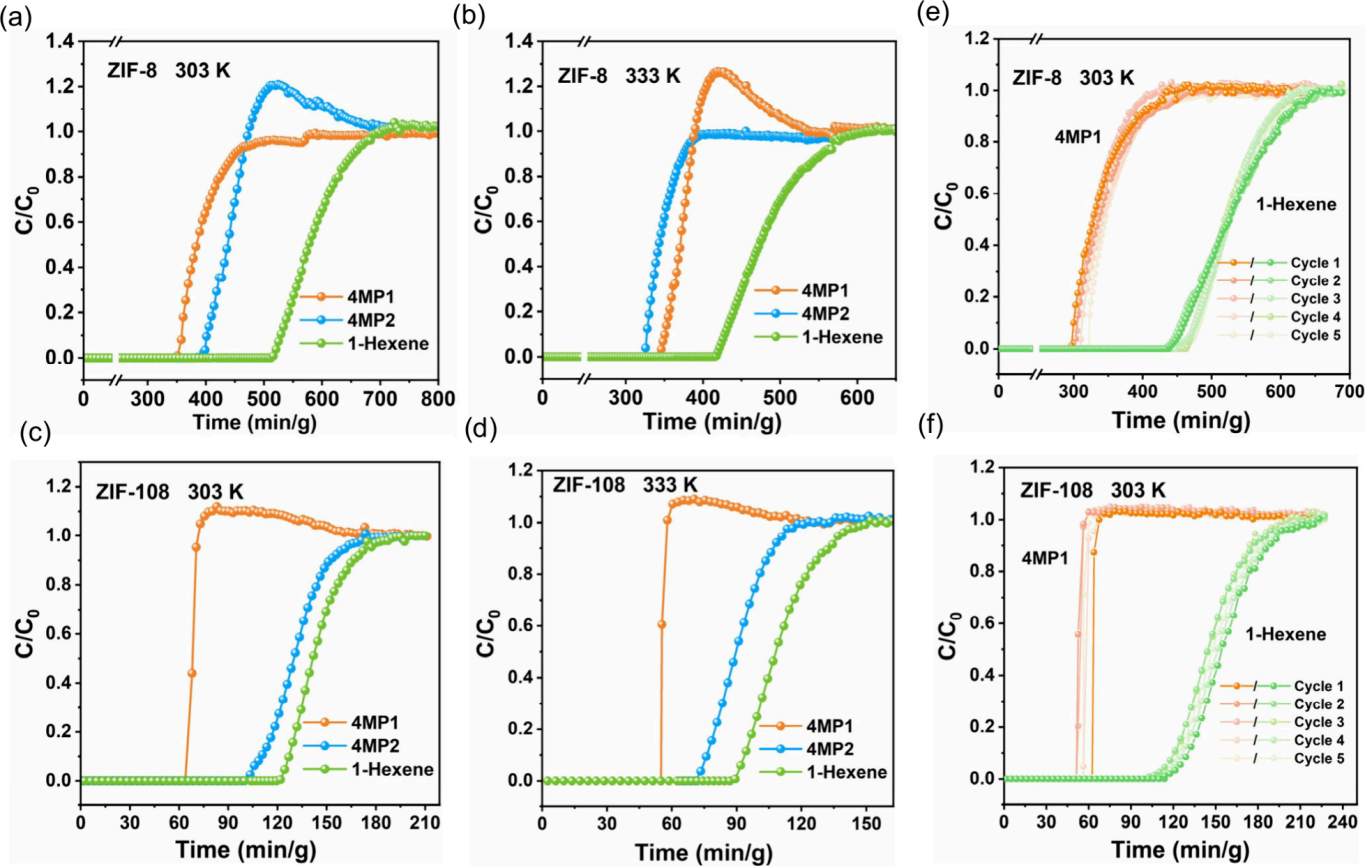
Dynamic breakthrough curves of 4MP1/4MP2/1-Hexene (18/1/1) at 303 K (a) and 333 K (b) on ZIF-8 with a flow rate of 1.0 mL/min. Dynamic breakthrough curves of 4MP1/4MP2/1-Hexene (18/1/1) at 303 K (c) and 333 K (d) on ZIF-108 with a flow rate of 1.0 mL/min. Cycling dynamic breakthrough curves of 4MP1/1-Hexene (18/1) at 303 K on ZIF-8 (e) and ZIF-108 (f).

Encouraged by the superior thermodynamic-kinetic adsorption performance and excellent stability, dynamic breakthrough experiments of various vapor mixtures were conducted to evaluate the actual purification performance of 4MP1. As shown in Figure S13, the separation of the 4MP1/4MP2 (18/1) and 4MP1/1-Hex (18/1) mixtures can be achieved on ZIF-8. However, the slowly rising breakthrough curves reflect the slow diffusion rates of these components in ZIF-8, which decreases separation efficiency and impedes the production of high-purity 4MP1, especially for mixtures of 4MP2 and 4MP1 with extremely low diffusion rates. The productivity of high-purity 4MP1 (>99.9%) during the separation process of 4MP1/1-Hex on ZIF-8 at 298 K was 0.740 mmol/g, while the productivity of 4MP1 from 4MP1/4MP2 was merely 0.180 mmol/g. Conversely, ZIF-108, with its larger pore window, enables highly efficient separation of both 4MP1/4MP2 and 1-Hex/4MP1 mixtures, characterized by rapidly ascending breakthrough curves (Figure S14). The productivities of high-purity 4MP1 from the 4MP1/1-Hex and 4MP1/4MP2 mixtures were 0.779 and 0.456 mmol/g, respectively. The outstanding separation performance of binary vapor mixtures prompted us to investigate the purification performance of 4MP1 from a simulated industrial mixture (4MP1/4MP2/1-Hex, v/v/v, 18/1/1). Similarly, the breakthrough curves of 4MP1 from ternary vapor mixtures (Figure 3a,b) on ZIF-8 exhibit slow ascents, yielding low productivity of high-purity 4MP1, only 0.168 and 0.061 mmol/g at 303 and 333 K, respectively. Benefiting from the high diffusion rates on ZIF-108, efficient separation of 4MP1/4MP2/1-Hex was accomplished (Figure 3c,d), with the productivity of high-purity 4MP1 significantly exceeding that on ZIF-8, reaching 0.458 and 0.241 mmol/g at 303 and 333 K, respectively. Detailed results of the breakthrough experiments are outlined in Tables S5–S8. Cycling breakthrough experiments of 4MP1/1-Hex were performed to investigate the cycling performance (Figure 3e,f). No significant decay in breakthrough time was observed during the cycling process, indicating a robust cycling stability.

**Figure 4:**
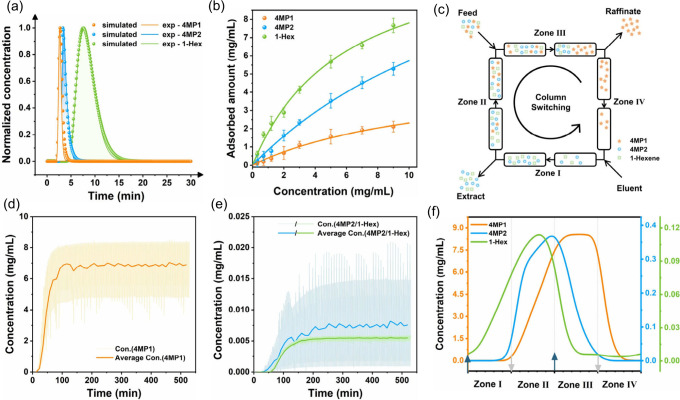
Experimental and simulated elution profiles of 4MP1, 4MP2, and 1-Hexene on ZIF-108 column (a). The liquid adsorption isotherms of 4MP1, 4MP2, and 1-Hexene on ZIF-108 (b). The scheme of SMB process (c). Concentration profiles of 4MP1, 4MP2, and 1-Hexene in the raffinate from a ZIF-108 column during the SMB simulation process, including detailed enlargements of 4MP2 and 1-Hexene concentrations at a feed concentration of 10 g/L (e). Internal profile obtained at the middle of the switching time at cyclic steady state at the feed concentration of 10 g/L.

The enhanced thermodynamic-kinetic adsorption separation performance and robust stability of ZIF-108 inspire us to explore its application potential in the SMB process. To verify the feasibility of ZIF-108 in SMB technology, we first conducted single-component separation through single-column pulsed liquid chromatography (Figure S15). As shown in Figure 4a, smooth and sharp peaks of 4MP1, 4MP2, and 1-Hex were observed on the ZIF-108 column, suggesting the potential for effective separation of these components in the liquid phase. The retention time of 4MP1 was only 2.6 min, reflecting the weakest affinity and lowest uptake for 4MP1 on ZIF-108. Despite the weaker affinity of ZIF-108 for 1-Hex compared to 4MP2, the greater saturation adsorption capacity of 1-Hex leads to a longer retention time for 1-Hex (7.4 min) than 4MP2 (3.4 min). The pore structure of the ZIF-108 column was characterized using the chromatographic adsorption method, with total porosity (ε_t_, 0.45) determined by the chromatographic tracer technique, as depicted in Figures S16 and described by eq 5, while internal porosity (ε_p_, 0.23) was assessed via the N_2_ adsorption isotherm at 77 K (eq 6a), and external porosity was subsequently calculated using eq 6b (see details in Supporting Information, Table S9). The liquid-phase adsorption isotherms of 1-Hex, 4MP1, and 4MP2 were also collected at 303 K (Figure 4b). ZIF-108 demonstrated a faster and sharper isotherm for 1-Hex compared to those of 4MP2 and 4MP1, highlighting the discrepancy in diffusion rates among the adsorbates within ZIF-108. The Henry’s selectivities of 1-Hex/4MP1 and 4MP2/4MP1, calculated based on the Langmuir equation, were 10.74 and 4.13, respectively (Table S10), further underscoring the potential for purifying 4MP1 from the liquid phase. To further investigate the kinetic adsorption behavior of these components in the liquid chromatography, we calculated the mass transfer coefficients (MTCs) through moment analysis.^45,46^ As displayed in Figure S17 and Table S9, the mass transfer coefficients follow the trend of 1-Hex > 4MP2 > 4MP1, consistent with the kinetic vapor adsorption results. Based on the parameters obtained (Table S9), the elution profiles were simulated. The simulated results closely matched the experimental data in Figure 4a, exhibiting good agreement in both peak shapes and positions with only minor discrepancies due to baseline shifts. This validation confirms the reliability of the parameters derived in this study, which are crucial for SMB simulations and operation design.

The complete simulation of the SMB process using the derived parameters was performed to assess the potential of ZIF-108 for SMB applications. As depicted in Figure 4c and Figure S18, the classical four-zone process was utilized, with each zone comprising two columns. The feedstock for the SMB consisted of an iso-octane solution with specific concentrations of 4MP1, 4MP2, and 1-Hexene, with pure iso-octane serving as the eluent. First, we applied the triangular theory (eqs 22–27) to calculate the operational ranges, including dimensionless flow rates in zone II and zone III (*m*_2_ and *m*_3_), for achieving complete separation of 1-Hexene/4MP1 and 4MP2/4MP1 mixtures under different feed concentrations. As noted in Figure S19, the operational range for complete separation narrows with increasing feed concentration. Additionally, the operational range for separation of 4MP1 and 4MP2 is entirely encompassed within that for separation of 1-Hexene and 4MP1. This is due to the lower separation selectivity for 4MP1 and 4MP2, which complicates their separation compared to those of 1-Hexene and 4MP1. Consequently, the operational range must be selected within the intersection of these two ranges. To minimize the entry of 4MP1 into the extraction phase, the dimensionless flow rate in zone I (*m*_1_) must be less than the Henry’s coefficient of 4MP1 (*H*_4MP1_). Additionally, for complete elution of 4MP2 and 1-Hexene, the dimensionless flow rate in zone IV (*m*_4_) should exceed the Henry’s coefficient of 1-Hex (*H*_1-Hex_), the most difficult component to elute. Considering these factors, we determined the optimal operating points, calculated the flow rates and switch times for each zone, and conducted simulations for the SMB process to separate 1-Hex/4MP2/4MP1. The specific operational parameters are detailed in Table S11. As shown in Figure 4d,e and Figures S20–S24, as feed concentration increases, the production capacity of the separation column improves, yet its anti-interference capability declines, resulting in a marginal reduction in the product purity. Specifically, at feed concentrations of 10, 20, and 30 g/L, the purity of 4MP1 in the raffinate phase reaches 99.8%, 99.5%, and 99.6%, respectively, with corresponding recovery rates of 96.5%, 97.4%, and 93.7%. Concentration distribution profiles within the adsorption column (Figure 4f and Figure S25) reveal that 4MP2 and 1-Hex are preferentially adsorbed, with only minor amounts reaching Zone III. Moreover, 4MP1 predominantly accumulates in Zone III, with a minimal presence in Zone I, and is chiefly carried out by the eluent. Its partial presence in Zone II is likely due to the higher 4MP1 content in the feedstock. Overall, employing ZIF-108 as an adsorbent in the SMB process facilitates the efficient separation of the ternary mixture of 1-Hexene/4MP2/4MP1, and high-purity 4MP1 can be obtained. The detailed purification performance of 4MP1 in the SMB process is outlined in Table S12.

**Figure 5:**
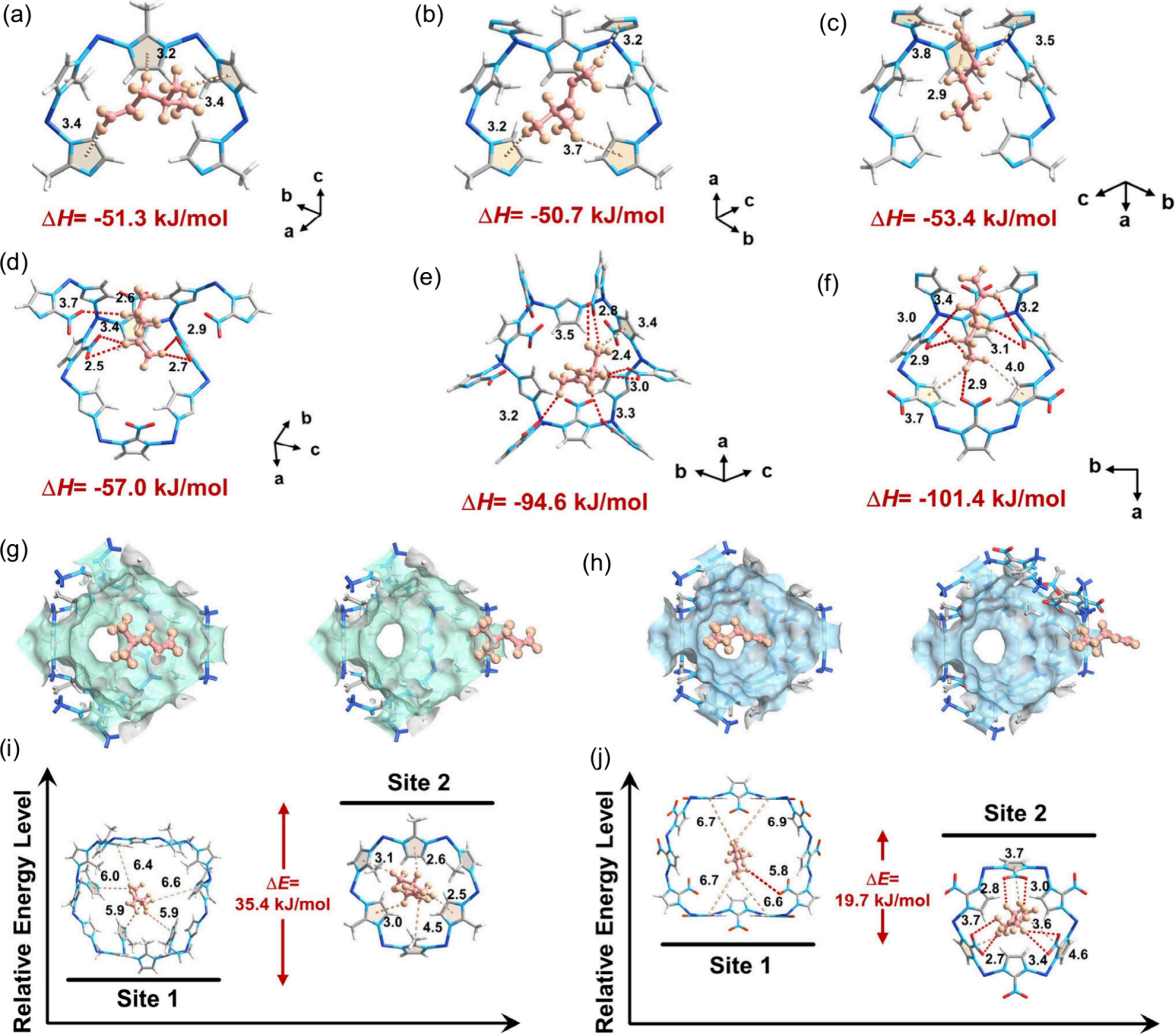
Calculated binding sites of 4MP1 (a), 4MP2 (b), and 1-Hexene (c) on ZIF-8. Calculated binding sites of 4MP1 (c), 4MP2 (d), and 1-Hexene (e) on ZIF-108. Schematic diagrams of 4MP1 molecules navigating through 1D channel of ZIF-8 (g) and ZIF-108 (h). 4MP1 interacting with framework at different sites and their corresponding energy levels in ZIF-8 (i) and ZIF-108 (j). Light pink dotted line represents C–H···π interactions and burgundy dotted line represent C–H···O interactions.

To gain deeper insights into the adsorption mechanism of these components in ZIF-8 and ZIF-108, we performed DFT calculations. As presented in Figure 5a–c, 4MP1, 4MP2, and 1-Hex are primarily located in the pore cavity of ZIF-108 and are mainly stabilized by C–H···π interactions formed by imidazole rings from the surrounding ligands, with the distances ranging from 2.9 to 3.8 Å. Notably, the binding energies of 4MP1, 4MP2, and 1-Hex on ZIF-8 are very similar, recorded at 51.3, 50.7, and 53.4 kJ/mol, respectively. These values correspond well with the trends observed in experimental measurements of adsorption enthalpies, suggesting similar thermodynamic affinities for these components on ZIF-8. For ZIF-108, in addition to the strong C–H···π interactions provided by the imidazole rings, the polar nitro groups on the ligands exert multiple C–H···O interactions (2.4–3.5 Å) toward these components (Figure 5d–f). Due to their significantly longer lengths, 1-Hex and 4MP2 exhibit more and stronger interactions with the framework compared to 4MP1. The binding energies of 1-Hex, 4MP2, and 4MP1 were calculated to be 101.4, 94.6, and 57.0 kJ/mol, respectively, significantly higher than those obtained in ZIF-8, further indicating the enhanced affinities and thermodynamic selectivity of these components in ZIF-108. To analyze the intrinsic reasons for the differences in adsorption affinities, the surface electrostatic potentials of different guest molecules and the MOF framework were calculated (Figures S26 and S27). Despite the similar structures of 4MP1 and 4MP2, the positions of the C=C bond cause variations in their electrostatic potential distributions. In 4MP2, the C=C bond, acting as an electron-withdrawing group, is centrally located, giving the hydrogens on both methyl ends a positive potential. In 4MP1, the C=C bond is at the end, resulting in a positive potential on only one methyl end. The pores of ZIF-8 are primarily composed of 2-methylimidazole, where the methyl group on the imidazole ring acts as an electron-donating functional group with a positive potential, slightly reducing the electronegativity of the imidazole ring. This weakens the interaction with 4MP2, which has positive potentials at both ends, leading to poor recognition of both 4MP1 and 4MP2. In ZIF-108, the replacement of the methyl group with a nitro group, a strong electron-withdrawing group, significantly increases the electronegativity in the pore channels. This enhances the interaction with 4MP2, which has positive potentials at both ends while potentially causing repulsion with the negatively charged end of 4MP1. Thus, ZIF-108’s pore channels enhance the differential affinity for 4MP2 and 4MP1, improving the selective adsorption of 4MP2. For the linear olefin 1-hexene, the longer molecular length compared to those of 4MP1 and 4MP2 means that ZIF-108, with its smaller pore cavity size, exhibits stronger confinement for 1-hexene compared to the larger cages of ZIF-8 and the shorter molecules 4MP1 and 4MP2, thereby achieving selective adsorption of 1-Hex. To further analyze the diffusion behavior of these components in ZIF-8 and ZIF-108 at the molecular level, MD simulations were conducted. To better elucidate the impact of the pore structure on diffusion, the adsorption behavior of 4MP1, which has the slowest diffusion rate, was analyzed. Energy fluctuations during the diffusion of 4MP1 within the MOF pore channels were recorded (Figure S28). The energy minimum for 4MP1 occurred at the center of the cavity, while the energy maximum was recorded when the molecule was positioned in the pore window. As shown in Figure 5g,h, when 4MP1 was located in the center of pore cavity of ZIF-8, the interactions between 4MP1 and the framework were very weak. However, as 4MP1 diffused to the narrow pore window, much stronger interactions were formed, leading to a high diffusion energy barrier (35.4 kJ/mol). In contrast, the slightly larger pore window in ZIF-108 promotes the diffusion of 4MP1, resulting in a lower diffusion energy barrier (19.7 kJ/mol) (Figure 5i,j). The discrepancy in the diffusion energy barrier between ZIF-8 and ZIF-108 underscores the effective enhancement of kinetic diffusion through pore window size regulation.

## Conclusion

In summary, the study demonstrates the capability of ZIF-108, a strategically engineered metal–organic framework, to significantly enhance the separation efficiency of 4-methyl-1-pentene from its isomers through simultaneous optimization of its thermodynamic and kinetic properties. By adjusting the pore architecture to feature narrower cavities and larger windows, ZIF-108 not only achieves higher molecular affinities but also facilitates greater diffusion rates compared with the traditional ZIF-8. The exceptional performance of ZIF-108 is validated through a range of experiments, including breakthrough curves and SMB simulations, supported by DFT calculations and MD simulations, which elucidate the molecular interactions contributing to the improved performance. This study highlights the potential of tailored pore engineering in MOFs to revolutionize separation processes in the chemical industry, especially for mixtures where traditional methods fall short. The robust stability and excellent regeneration capabilities of ZIF-108 further underscore its suitability for industrial applications, paving the way for more energy-efficient and effective separation technologies.

## Supporting Information Available

Synthesis method of MOFs, characterizations details, adsorption tests and breakthrough experiments, elution profiles measurement, pore structure measurement, liquid-phase adsorption measurement, SMB simulation details, DFT calculation and MD simulation details, dimensional size of adsorbate, XPS spectrum, FT-IR results, SEM images and EDS mapping results, N_2_ adsorption isotherms at 77 K, pore size distributions, adsorption isotherms, DSC results, PXRD patterns, TGA results, breakthrough curves, scheme of pulse elution profiles, the relationship between the retention time and *L*/*u* on ZIF-108 column, *HETP* of 4MP1, 4MP2 and 1-Hex of ZIF-108 column, scheme of SMB process and the concentration profile in ZIF-108 column, schematic diagram of the operational range, the concentration of components in ZIF-108 during SMB process, concentration profiles of components, surface electrostatic potential of guest molecules, surface electrostatic potential of ZIF-8 and ZIF-108, energy variation of 4MP1 during the diffusion pathway, crystal data and structure refinement results, BET surface areas, pore volumes and pore sizes of samples, summary of gas uptake and selectivity in various materials, kinetic diffusion parameters, calculation results of breakthrough experiments, design and model parameters for SMB, parameters and selectivity of liquid chromatographic column of ZIF-108, operating parameters of SMB, calculation results of SMB process (PDF)
