# Supplementary material for: Planar Group Functionalization of Quasi-Discrete Pores in Metal–Organic Frameworks for Enhanced Isomeric Separation in Simulated Moving Bed Processes
Source: ACS Cent Sci. 2024 Aug 27;10(10):1861–70. doi: 10.1021/acscentsci.4c00876 (PMC11503489; doi:10.1021/acscentsci.4c00876)
Supplement: Supplementary file 1 — oc4c00876_si_001.pdf [file oc4c00876_si_001.pdf]

Planar Group Functionalization of Quasi-Discrete Pores in Metal-Organic Frameworks for Enhanced Isomeric Separation in Simulated Moving Bed Processes

Zhe Chu,[1] Jiaqi Li, [1] Fuqiang Chen,[1,] Yifeng Cao,[2] Lihang Chen,[2] Feng Zhou,[3] Huixia Ma,[3] Qiwei Yang,[1,2] Zhiguo Zhang,[1,2] Kai Qiao,[3,*] Qilong Ren,[1,2] and Zongbi Bao[1,2,*]*

[1], Key Laboratory of Biomass Chemical Engineering of ministry of Education, College of Chemical and Biological Engineering, Zhejiang University,866 Yuhangtang Road, Hangzhou 310058, P. R. China*

[2] Institute of Zhejiang University-Quzhou, 99 Zheda Road, Kecheng District, Quzhou 324000, P. R. China

[3] SINOPEC (Dalian) Research Institute of Petroleum and Petrochemicals Co., Ltd., 96 Nankai Street, Lvshunkou District, Dalian 116045, P.R. China

* Email: chen_fq@zju.edu.cn, qiaokai.fshy@sinopec.com, baozb@zju.edu.cn

Experimental section

Materials

All commercial chemicals were analytical reagents and used without further purification. $Zn(NO_3)_2\cdot6H_2O$ (99%), $Zn(OAc)_2\cdot2H_2O$, 2-methylimidazole (99%), 2-nitroimidazole (99%), 4-methyl-1-pentene (97%), 4-methyl-2-pentene (98%), 1-Hexene (99%), triisopropylbenzene (99%), isooctane (99%), dimethylformamide(DMF, 99%) and methanol(MeOH, 99%) were purchased from TCI Co. Ltd. Ultrahigh purity grade He (99.999%), CO_2 (99.999%) and N2 (99.999%) were purchased from Jingong Co., Ltd. (China).

Synthesis of ZIF-8

ZIF-8 was synthesized based on the previously reported method with minor modifications.[1] $Zn(NO_3)_2\cdot6H_2O$ (1173 mg) and 2-methylimidazole (2595 mg) were dissolved in MeOH (120 mL) and stirred for 30 minutes. Then the suspension was centrifuged and washed 3 times with MeOH.

Synthesis of ZIF-108

ZIF-108 was synthesized based on the previously reported method with minor modifications.[2] $Zn(OAc)_2\cdot2H_2O$ (636 mg) and 2-nitroimidazole (656 mg) were dissolved in DMF (56 mL) and stirred for 120 minutes. Then the suspension was centrifuged and washed 3 times with DMF.

Preparation of chromatographic column of ZIF-108

ZIF-108 was activated under vacuum at 120 °C for 12h, and then the suspension was prepared with isooctane, which was dropped into an empty 0.46×25 cm^2 column tube. The column was pressed with a liquid-phase pump at flow rate of 2 mL /min and pressure of 15 MPa. After the top end of the column produces interspace continue dropping in the suspension and again stamping with the liquid phase pump, repeating the above until the top end of the column does not produce interspace.

Sample Characterization

Powder X-ray diffraction (PXRD) patterns of the samples were collected on a X'Pert diffractometer (Panalytical Corp., Netherlands) using Cu Kα radiation (λ = 1.542 Å) radiation at 40 kV/40 mA from 5° to 60° (2θ angle range) in a step of 0.02°. The single crystal X-ray diffraction of the as-synthesized was performed at 193 K using a Bruker D8 VENTURE PHOTON II system equipped with a Liquid Metal source MetalJet (Ga Kα, λ = 1.34139 Å). The Brunauer-Emmett-Teller (BET) specific surface areas were determined by N_2 adsorption-desorption isotherms at 77 K, which were performed on 3 Flex (Micromeritics) volumetric adsorption analyzer. Brunauer-Emmett-Teller (BET) specific surface areas were assessed according to the BET equation with the pressure range of $0.05 < p/p_0 < 0.20$ for calculations. The thermal gravimetric analysis (TGA) was performed in TA-Q500 (TA Instruments) with heating rate of 10 °C min-1 under N_2 atmosphere from 50 to 800 °C.

Vapor adsorption measurements

The samples of ZIF-8 and ZIF-108 were degassed at 393 K for 24 h under ultrahigh vacuum prior to vapor adsorption measurements of 4MP1, 4MP2 and 1-Hex. The single component isotherms of 4MP1, 4MP2, and 1-Hex were measured at 303 and 333 K on a BELSORP-max II (BEL Japan, Inc.) equipped with a vapor dosing bottle. Each sample tube was subsequently immersed in a temperature-controlled heating mantle that surrounded most of the sample tube. The manifold of the instrument itself including the vapor dosing bottle was heated to 323 K and kept at this temperature for all these single-component measurements.

Vapor kinetic adsorption measurements

The kinetic adsorption tests of 4MP1, 4MP2 and 1-Hex was measured on a BELSORP-max II (BEL Japan, Inc.) automated volumetric sorption analyzer and fitted automatically with BEL-Master software. About 100 mg sample was degassed at 393 K under ultrahigh vacuum for 24 h before the experiments. The kinetic adsorption characteristics were measured when the adsorption equilibrium data were collected at 303 K and 0.45 kPa. After the

adsorbate gas was introduced into the adsorption system at a given dose, the changes of gas pressure and 4MP1, 4MP2 and 1-Hex uptake with time were recorded and converted into the transient normalized uptake as a function of time. The kinetic selectivity can be defined as the ratio of the two vapors' diffusion time constants. Diffusion time constants (D_c/r_c^2) were calculated by the short-time solution of the diffusion equation assuming a step-change in the gas-phase concentration with the following micropore diffusion model. The microporous adsorbent particle exposed to a step-change in sorbate concentration at the external surface of the particle at time zero. Heat transfer is assumed to be sufficiently rapid, relative to the sorption rate.

$$\frac{m_t}{m_\infty} \approx \frac{6}{\sqrt{\pi}}\sqrt{\frac{D_c t}{r_c^2}} - 3\frac{D_c t}{r_c^2} \quad \left(\frac{m_t}{m_\infty} < 0.85\right) \tag{1}$$

where D_c is the intracrystalline diffusivity, m_t is the adsorbed at time t, m_∞ is the adsorbed amount at equilibrium, and r_c is the radius of the equivalent spherical particle.

Differential Scanning Calorimetry (DSC)

Adsorption heats of 4MP1, 4MP2, and 1-Hex on ZIF-8 and ZIF-108 were measured by differential scanning calorimetry (DSC) on STD 650 (TA Instruments, Inc.). Runs at the thermobalance were performed by feeding a flow stream of 4MP1, 4MP2, or 1-Hex diluted with N_2 onto about 10 mg of evacuated sample at 303 K. Prior to measurements, the baseline was monitored under dry nitrogen flow at the same temperature of 303 K, and then a N_2 gas flow was introduced by bubbling the carrier gas in a saturator containing single-component liquid of 4MP1, 4MP2, and 1-Hex at a given temperature and the DSC signal were recorded to obtain the heat of adsorption. The heat of adsorption is calculated by the follow equation:

$$\Delta H_{\mathrm{ads}} = \frac{\Delta H_{\mathrm{Total}}}{\Delta m_{\mathrm{ads}} \times M_{\mathrm{guest}}} \tag{2}$$

where ΔH_{ads} is adsorption heat (kJ/mol), ΔH_{Total} is sum of the change in heat flow during adsorption, Δm_{ads} is the mass change of the adsorbent, and M_{guest} is the relative molecular weight of guest.

Breakthrough experiments

In a typical experiment, of activated ZIF-8 (0.9 g) or ZIF-108 (0.8 g) sample was packed into a stainless steel HPLC column (100 mm I.D.×4.6 mm). The column packing was conducted in glove-box filled with Ar. A stream of N_2 flow (20 mL/min) was introduced into the column to further purge the sample at 393 K for 24 h prior to measuring the vapor breakthrough experiments. A mixture of ternary 4MP1, 4MP2, and 1-Hex was loaded into a glass bubbler that connected to the sample column and N_2 cylinder, respectively. N_2 (99.999%) was flowed through the bubbler at a rate of 10 mL/min, which was controlled by a mass flow controller. The composition of the ternary components in the bubbler was adjusted until the mixture ratio of 18/1/1 was achieved in the vapor phase as detected by the gas chromatograph (GC). The mixture from the bubbler was carried by N_2 flow through the sample column to the GC with a rate of 0.5 mL/min. The temperature of the sample column was controlled through an oil bath at 303 and 333 K, respectively, to evaluate the 4MP1 separation performance on the ZIF-8 and ZIF-108 samples at various temperatures. The binary mixture of 4MP1/4MP2 or 4MP1/1-Hex was also introduced with a constant flow rate of 0.5 mL/min and the sample column was controlled through an oil bath at 303 and 333K, respectively. The bubbler containing binary isomers was controlled at specific temperature to make the partial pressure of 4MP1, 4MP2 and 1-Hex component equal to that of the ternary mixtures. The outlet vapor from the sample column was monitored using a GC-2010 Pro (SHIMADZU) gas chromatography with a flame ionization detector (FID). The vapor mixture was separated by a capillary column (PONA-5, Φ0.25×50m) at 343 K. After every breakthrough experiment, the sample column was activated and regenerated with a N_2 flow at 393 K for 72-96 h.

The adsorbed amounts of vapor i (q_i) is calculated from the breakthrough curves using the following equation:

$$q_i = \frac{F_i \times P_i \times \left(t_0 - \int_0^{t_0} \frac{C_{(t)}}{C_0} dt\right)}{V_m \times m} \quad (3)$$

F_i is the flow rate of component i, mL/min; t_0 is the breakthrough equilibrium time, min; $\int_0^{t_0} \frac{C_{(t)}}{C_0} dt$ is the area enclosed by the breakthrough curve of component i from 0 to moment t with the x-axis; V_m is the molar volume of the vapor, this value is 22.4 mL/mmol; *m* is the mass of adsorbents packed in the filled column, g.

The high-purify production of 4MP1 is calculated from the breakthrough curves using the following equation:

$$q_i = \frac{F_{\text{Vapor,4MP1}} \times P_{4MP1} \times \int_0^{t_{4N}} \frac{C_{(t)}}{C_0} dt}{V_{\text{Vapor,4MP1}} \times m} \quad (4)$$

$F_{\text{Vapor, 4MP1}}$ is the flow rate of 4MP1 vapor, mL/min; t_{4N} is the time of 4MP1 purity exceeding 99.99%, min; $\int_0^{t_{4N}} \frac{C_{(t)}}{C_0} dt$ is the area enclosed by the breakthrough curve of 4MP1 from 0 to moment t_{4N} with the x-axis; $V_{\text{Vapor, 4MP1}}$ is the molar volume of the vapor, this value is 22.4 mL/mmol; *m* is the mass of adsorbents packed in the filled column, g.

Elution profiles measurement

The elution profiles measurement were performed using the procedure shown in Figure S9, including a Quiksep P0010 HPLC PUMP, R1-201H Refractive Index Detector and a six-way injection valve. The ZIF-108 liquid chromatography column was flushed with chromatographic grade iso-octane at 1 mL/min until the detector baseline was equilibrated, and then 10 µL of 4MP1, 4MP2, and 1-Hex were injected using the manual injector respectively.

Pore structure Measurement

The total porosity can be evaluated from the retention time of a non-retained tracer substance. triisopropylbenzene is normally considered to be non-adsorbed by reversed phase chromatography. The total porosity is calculated according to

$$\mathrm{t}_0 = \varepsilon_t \frac{L}{u} \quad (5)$$

where, *u* is the superficial velocity of mobile phase; t_0 is the retention time of uracil; ε_t is the total porosity of ZIF-108 column; *L* is the length of the column.

Experiments were performed at five different flow rates from 0.5 to 2.5 mL/min. The retention times of triisopropylbenzene were plotted against the inverse superficial velocity of mobile phase. As shown in Figure S10, ε_t was then determined from the slope of the straight line. The value of ε_t was found to be 0.45 with correlation coefficients of R^2 = 0.999, respectively.

Internal pore volume was measured by N_2 adsorption isotherm at 77 K on 3-Flex (Micromeritics) volumetric adsorption analyzer, V_p= 0.62 cm^3/g. The external porosity is calculated according to

$$\varepsilon_p = \frac{V_p}{1-\varepsilon} \quad (6a)$$

$$\varepsilon_t = \varepsilon - (1-\varepsilon)\varepsilon_p \quad (6b)$$

where, ε_p is the internal porosity; ε is the external porosity; V_p is internal pore volume.

Moment balance

The moment analysis is one of the most basic strategies to estimate the transport coefficients of column. Parameter estimation by the method of moment analysis has been covered in detail previously[3-4]. The mean retention time is related to the linear adsorption equilibrium constant, while the spread of the response peak is related to the combined effects of axial dispersion and mass transfer resistance. The corresponding equations of the first and second moments for the lumped pore diffusion model are:

$$\mu_1 = \frac{L}{u_{\text{int}}}(1+G) \quad (7a)$$

$$\mu_2 = 2\left(\frac{L}{u_{\text{int}}}\right)^2\left[G^2\frac{\varepsilon}{1-\varepsilon}\frac{r_{\text{p}}u_{\text{int}}}{3k_{\text{eff}}L} + \frac{D_{ax}}{u_{\text{int}}L}(1+G)^2\right] \quad (7b)$$

$$G = \frac{1-\varepsilon}{\varepsilon}\left[\varepsilon_{\text{p}} + (1-\varepsilon_{\text{p}})H\right] \quad (7c)$$

The expression for the height equivalent to a theoretical plate (*HETP*) derived from the second moment analysis is:

$$N = 5.545\left(\frac{t_{\text{R}}}{W_{\text{h}}}\right)^2 \quad (8a)$$

$$HETP = \frac{L}{N} = \frac{\mu_1 L}{\mu_2} = \frac{2D_{ax}}{u_{\text{int}}} + 2\left(\frac{G}{1+G}\right)^2 \varepsilon\frac{\varepsilon}{1-\varepsilon}\frac{r_{\text{p}}}{3k_{\text{eff}}}u_{\text{int}} \quad (8b)$$

$$MTCs = \frac{6k_{\text{eff}}}{r_{\text{p}}} \quad (8c)$$

in the above equations, u_{int} is linear velocity of the fluid phase; μ_1 (min) is first moment, μ_2 is second moment, N is the theoretical plate number, W_{h} (min) is the peak width at half height, and t_{R} (min) is the retention time. Equation (5b) contains two model parameters of interest, the axial dispersion coefficient (D_{ax}) and the effective mass transfer coefficient (k_{eff}) which could be considered as constants in the range of flow rates. N is number of theoretical plates. *HETP* is the height equivalent to a theoretical plate. The second moment analysis is based on the assumption that both D_{ax} and k_{eff} are not influenced by velocity. r_{p} is adsorbent particle size. Therefore, Equation (5b) implies that the two model parameters of interest can be determined by a non-linear least-square regression due to the relationship between *HETP* and the interstitial fluid velocity. *MTCs* are the mass transfer coefficients. Furthermore, the simple procedure outlined above can be used to obtain reasonable estimates of Henry constants also.

As seen in Figure S11, the *HETP* was linearly related to u_{int}, which was caused by the small contribution of molecular diffusion to axial diffusion, which consists of molecular and eddy current diffusion, as shown in Equation (6):

$$D_{\text{ax}} = \gamma D_{\text{m}} + \gamma d_{\text{p}}u_{\text{int}} \quad (9)$$

where D_{m} is molecular diffusion, γ is distortion factor which is a constant related to the packing effect of the column. Since d is relatively small, the above equation can be approximated as a linear relation:

$$D_{\text{ax}} \approx \gamma d_{\text{p}}u_{\text{int}} \quad (10)$$

For 4MP1, 4MP2 and 1-Hex, the axial coefficient D_{ax} should be of the same magnitude due to the neglect of molecular diffusion, so the parameters obtained are listed in Table S9.

Liquid-phase adsorption measurement

Adsorption equilibrium isotherm describes the distribution of solutes between the mobile and the stationary phases, and it provides the core information for the design of an SMB separation. Competitive Langmuir is the most commonly applied isotherm model for SMB separation process[5]. The model is based on the assumption that the forces of interaction between adsorbed molecules are negligible, and the different solutes adsorb to and compete for the same sites. The isotherm model is described by Equation (8)

$$q_{\text{i}} = \frac{H_{\text{i}}c_{\text{i}}}{1+\sum b_{\text{i}}c_{\text{i}}} \quad (11)$$

where H_{i} is Henry constant and b_{i} is equilibrium constant.

For single component, Equation (9) becomes:

$$q_{\text{i}} = \frac{H_{\text{i}}c_{\text{i}}}{1+b_{\text{i}}c_{\text{i}}} \quad (12)$$

In this work, the single component and binary competitive nonlinear adsorption equilibrium isotherm are measured by frontal chromatography method[6], which is tedious but accurate. The column was pre-equilibrated with the mobile phase (isooctane), then the adsorbent isooctane solution dissolved in mobile phase at a known concentration was continuously fed into the column at 0.5 mL /min and the effluent solution was simultaneously detected online by refractive detector. When the adsorption equilibrium was reached, the time at the peak of the first-

order derivative of the efflux curve was taken as the adsorption time t_e, and the adsorption amount of the ZIF-108 column at the concentration of this adsorbent was calculated by Equation (10)

$$q_{\mathrm{feed,i}} = \frac{c_{\mathrm{feed,i}}(t_{\mathrm{e}}v - V\varepsilon_{\mathrm{t}})}{V(1-\varepsilon_{\mathrm{t}})} \tag{13}$$

Where V (mL) is volume of column, and ε_t is total porosity, and $c_{\mathrm{feed,i}}$ (mg/mL) is the concentration of adsorbent feed, and $q_{\mathrm{feed,i}}$ is adsorption amount of the ZIF-108 column at the concentration of this adsorbent, and v is flow rate of feed.

Theory of simulation of elution profiles

The model of elution profiles, including a ZIF-108 column and pulse injector, was established by Aspen Chromatography to monitor the concentration curves at outlet of the column as a function of time. The mass of partial differential equations (PDEs), such as mass balance, momentum balance, and energy balance need to be solved in the elution profiles simulation. Aspen Chromatography uses the spatial discretization method to solve partial differential equations. Upwind Differencing Scheme 1 (UDS1) was selected with 20 nodes as the discretization method since it shows compatibility with accuracy and short simulation time. Then implicit Euler integration method is used to solve the differential equations.

Theory of simulation of SMB

The 4-zone SMB process, including 16 columns, was simulated by Aspen Chromatography to evaluate the performance of ZIF-108 adsorbent in separating 4MP1 from 4MP1/4MP2/1-Hex (18/1/1, m/m/m) mixtures.

The mass of partial differential equations (PDEs), such as mass balance, momentum balance, and energy balance need to be solved in the SMB simulation. Aspen Chromatography uses the spatial discretization method to solve partial differential equations. Upwind Differencing Scheme 1 (UDS1) was selected with 20 nodes as the discretization method since it shows compatibility with accuracy and short simulation time. Then implicit Euler integration method is used to solve the differential equations.

Mathematical modeling

Lumped pore model was used to describe the chromatography process[5], where axial dispersion and mass transfer resistance are taken into account. The mass balance equation in bulk mobile phase is written by Equation (11):

$$\frac{\partial c_{\mathrm{i,k}}}{\partial t} + u_{\mathrm{int,k}}\frac{\partial c_{\mathrm{i,k}}}{\partial x} + \frac{(1-\varepsilon)}{\varepsilon}\left[\varepsilon_{\mathrm{p}}\frac{\partial c_{\mathrm{p,i,k}}}{\partial t} + (1-\varepsilon_{\mathrm{p}})\frac{\partial c_{\mathrm{p,i,k}}}{\partial t}\right] = D_{\mathrm{ax,i}}\frac{\partial^2 c_{\mathrm{i,k}}}{\partial x^2} \tag{14}$$

where i are the species in the mixture, k is column number, $c_{\mathrm{i,k}}$ (mg/mL) is the concentration of component i in the mobile phase of column k, $c_{\mathrm{p,i,k}}$ (mg/mL) is average concentration in particle pore, $q_{\mathrm{i,k}}$ (mg/mL) is the average concentration in stationary phase in local equilibrium with $c_{\mathrm{p,i,k}}$, $u_{\mathrm{int,k}}$ (cm/min) is the interstitial velocity, $D_{\mathrm{ax,i}}$ (cm^2/min) is the axial dispersion coefficient, ε is the bed void-age of the column, ε_p is the internal porosity, x (cm), and t (min) are the space and time coordinates, respectively.

The mass transfer in particle is defined by the linear driving force model:

$$\varepsilon_{\mathrm{p}}\frac{\partial c_{\mathrm{p,i,k}}}{\partial_t} + (1-\varepsilon_{\mathrm{p}})\frac{\partial q_{\mathrm{i,k}}}{\partial t} = k_{\mathrm{eff,i}}\frac{3}{r_{\mathrm{p}}}(c_{\mathrm{i,k}} - c_{\mathrm{p,i,k}}) \tag{15}$$

where r_p (cm) is the average radius of the adsorbent particles, $k_{\mathrm{eff,i}}$ (cm/min) is the effective mass transfer coefficient, which is used to summarize both the internal and external mass transfer resistance effects. Initial conditions for each column are:

$$t = 0,\ c_{\mathrm{i,k}} = q_{\mathrm{i,k}} = c_{\mathrm{p,i,k}} = 0 \tag{16}$$

Danckwerts condition is frequently used at the inlet and outlet of each column as boundary conditions:

$$x = 0,\ D_{\mathrm{ax,i}}\frac{\partial c_{\mathrm{i,k}}}{\partial x} = u_{\mathrm{int,k}}\left(c_{\mathrm{i,k}} - c_{\mathrm{i,k}}^{\mathrm{in}}\right) \tag{17a}$$

$$x = L,\ \frac{\partial c_{\mathrm{i,k}}}{\partial x} = 0 \tag{18b}$$

where the superscript "in" refers to the inlet of streams, L (cm) is the length of column.

Node balance can be written as follows: at eluent node,

$$Q_1 = Q_\mathrm{D} + Q_4 \tag{18a}$$

$$c_\mathrm{i,k+1}^\mathrm{in} = \frac{Q_4 c_\mathrm{i,k}^\mathrm{out}}{Q_1} \tag{18b}$$

at extract node,

$$Q_2 = Q_1 - Q_\mathrm{Ex} \tag{19a}$$

$$c_\mathrm{i,k+1}^\mathrm{in} = c_\mathrm{i,k}^\mathrm{out} \tag{19b}$$

at feed node,

$$Q_3 = Q_2 + Q_\mathrm{F} \tag{20a}$$

$$c_\mathrm{i,k+1}^\mathrm{in} = \frac{Q_\mathrm{F} c_\mathrm{feed,\,i} + Q_2 c_\mathrm{i,k}^\mathrm{out}}{Q_3} \tag{20b}$$

at raffinate node,

$$Q_4 = Q_3 - Q_\mathrm{Ra} \tag{21a}$$

$$c_\mathrm{i,k+1}^\mathrm{in} = \frac{Q_4 c_\mathrm{i,k}^\mathrm{out}}{Q_1} \tag{21b}$$

where the superscript "out" denote the outlet of streams, $c_\mathrm{feed,i}$ (mg/mL) is the feed concentration, Q_1 (mL/min), Q_2 (mL/min), Q_3 (mL/min), and Q_4 (mL/min) are the flow rates of sections 1, 2, 3, and 4 of SMB, Q_Ra (mL/min), Q_Ex (mL/min), Q_D (mL/min), and Q_F (mL/min) are the flow rates of raffinate, extract, eluent, and feed, respectively.

Triangle theory

The successful operation of an existing SMB unit depends on the proper selection of the operating conditions: the flow rates in each section and the switching time. Developed in the frame of equilibrium theory, the triangle theory is the widely applied approach for SMB design[7], which neglects the effects of axial mixing and mass transfer resistances. Based on this theory, the operating conditions can be expressed in terms of dimensionless flow rates in each section:

$$m_\mathrm{j} = \frac{Q_\mathrm{j}^\mathrm{SMB} t_\mathrm{s} - V_\mathrm{col}\varepsilon_\mathrm{t}}{V_\mathrm{col}(1-\varepsilon_\mathrm{t})} \tag{22}$$

where $Q_\mathrm{j}^\mathrm{SMB}$ (mL/min) is the volumetric flow rate of each section, t_s (min) is the switching time, V_col (mL) is the volume of the column, m_j is dimensionless flow rate in each section, and ε_t is the total porosity of the column.

As for the nonlinear adsorption isotherm in this work, the boundaries for complete separation of two components feed mixture are given by the following set of inequalities:

The line *wf*:

$$[H_\mathrm{A} - \omega_1(1 + b_\mathrm{A}c_\mathrm{A})]m_2 + b_\mathrm{A}c_\mathrm{A}\omega_1 m_3 = \omega_1(H_\mathrm{A} - \omega_1) \tag{23}$$

The line *wb*:

$$[H_\mathrm{A} - H_\mathrm{B}(1 + b_\mathrm{A}c_\mathrm{A})]m_2 + b_\mathrm{A}c_\mathrm{A}H_\mathrm{A} m_3 = H_\mathrm{B}(H_\mathrm{A} - H_\mathrm{B}) \tag{24}$$

The curve *ra*:

$$m_3 = m_2 + \frac{\left(\sqrt{H_\mathrm{B}} - \sqrt{m_2}\right)^2}{b_\mathrm{B}c_\mathrm{A}} \tag{25}$$

The line *ab*:

$$m_2 = m_3 \tag{26}$$

The intersection points of each curve are as follows:

Point a:$(H_\mathrm{A}, H_\mathrm{A})$

Point a:$(H_\mathrm{B}, H_\mathrm{B})$

Point f:(ω_1, ω_1)

Point r: $\left(\frac{\omega_1^2}{H_\mathrm{A}}, \frac{\omega_1[\omega_2(H_\mathrm{A}-\omega_1)(H_\mathrm{A}-H_\mathrm{B}) + H_\mathrm{B}\omega_1(H_\mathrm{A}-\omega_1)]}{H_\mathrm{A}H_\mathrm{B}(H_\mathrm{A}-\omega_2)}\right)$

Point w: $\left(\frac{H_\mathrm{B}\omega_1^2}{H_\mathrm{A}}, \frac{\omega_1[\omega_2(H_\mathrm{A}-\omega_1)(H_\mathrm{A}-H_\mathrm{B}) + H_\mathrm{B}(H_\mathrm{B}-\omega_2)]}{H_\mathrm{B}(H_\mathrm{A}-\omega_2)}\right)$

Where 1 and 2 are parameters related to the composition and concentration of the raw material, which can be obtained by solving the following Eq (24):

$$(1 + b_{\mathrm{A}}c_A + b_{\mathrm{B}}c_B)\omega^2 - [H_{\mathrm{A}}(1 + b_{\mathrm{B}}c_B) + H_{\mathrm{B}}(1 + b_{\mathrm{A}}c_A)]\omega + H_A H_{\mathrm{B}} = 0 \tag{27}$$

the subscripts "A" and "B" represent the more retained component (4MP2 and 1-Hex) and the less retained component (4MP1), respectively.

SMB performance

The performance of the SMB process is analyzed using the following indicators: relative purity, recovery rate. They are used to compare different SMB runs. The relative purity (%) can be defined as the ratio between the mean concentration of single component and the total mean concentration of two components in each stream over a switch interval at steady state:

$$Purity_{\mathrm{A}} = \frac{C_{\mathrm{Ex,A}}}{C_{\mathrm{Ex,A}} + C_{\mathrm{Ex,B}}} \tag{28a}$$

$$Purity_{\mathrm{B}} = \frac{C_{\mathrm{Ra,B}}}{C_{\mathrm{Ra,A}} + C_{\mathrm{Ra,B}}} \tag{28b}$$

The recovery rate (%) is defined as the ratio of the amount of a particular component in corresponding product stream over the total amount of that component fed over a switch interval:

$$\text{Recovery rate}_{\mathrm{A}} = \frac{Q_{\mathrm{Ra}}C_{\mathrm{Ra,\ A}}}{Q_{\mathrm{F}}C_{\mathrm{feed,\ A}}} \tag{29a}$$

$$\text{Recovery rate}_{\mathrm{B}} = \frac{Q_{\mathrm{Ex}}C_{\mathrm{Ex,B}}}{Q_{\mathrm{F}}C_{\mathrm{feed,\ B}}} \tag{29b}$$

Dispersion-corrected density functional theory (DFT-D) calculations

All periodic DFT-D calculations were performed by the Materials Studio package.3. Vanderbilt-type ultrasoft pseudopotentials and the generalized gradient approximation (GGA) with the Perdew-Burke-Ernzerhof (PBE) functional and the double numerical plus d-functions (DNP) basis set were used for all structure geometry optimization calculations. A cutoff energy of 544 eV and a 2×2×1 k-point mesh was found to be enough for total energy to converge within 0.01 meV/atom. Full geometry optimizations were performed on the structures loaded with one 4MP1, 4MP2, or 1-Hex molecules. The static binding energy was calculated by the following equation:

$$E(\mathrm{B}) = E(\text{guest}) + E(\text{adsorbent}) - E(\text{adsorbent} + \text{guest}) \tag{30}$$

Molecular dynamics simulation

Structure optimization of the guest molecule and crystal cell were constructed respectively using the Geometry optimization module, choosing Universal force field and Smart algorithm, with van der Waals forces and electric fields based on atom calculations. The geometrically optimized guest molecules were placed into the cell and the relative potential energy was calculated separately along the diffusion route. The diffusion energy barrier was the difference between the highest and lowest potential energy of the diffusion route.[8-9] In this process, the maximum energy convergence threshold was 0.01 kacl·mol^{-1} and the maximum force convergence threshold was 0.05 kacl·mol^{-1}·Å^{-1}.

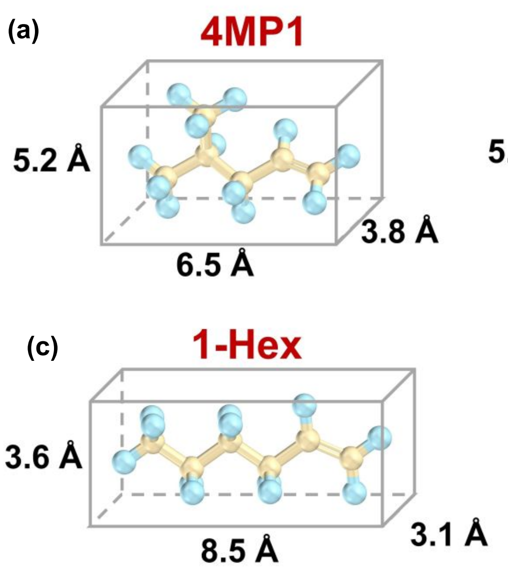

Figure S1. The dimensional size of (a) 4MP1, (b) 4MP2, and (c) 1-Hexene.

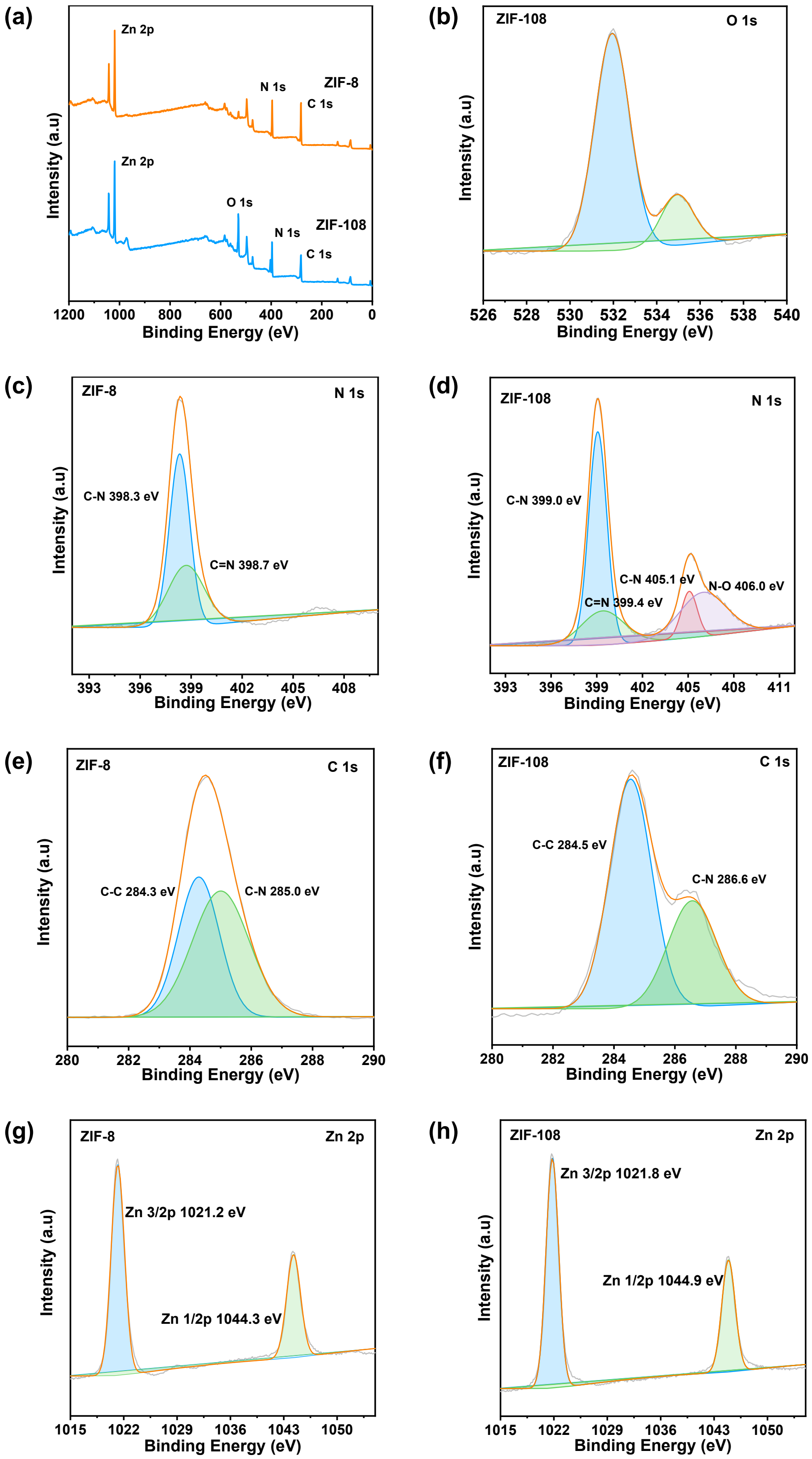

Figure S2. The XPS survey spectra on ZIF-8 and ZIF-108 (a). High-resolution O 1s spectrum of ZIF-108 (B); High-resolution N 1s spectrum of ZIF-8 (c) and ZIF-108 (d); High-resolution C 1s spectrum of ZIF-8 (e) and ZIF-108 (f); High-resolution Zn 2p spectrum of ZIF-8 (g) and ZIF-108 (h).

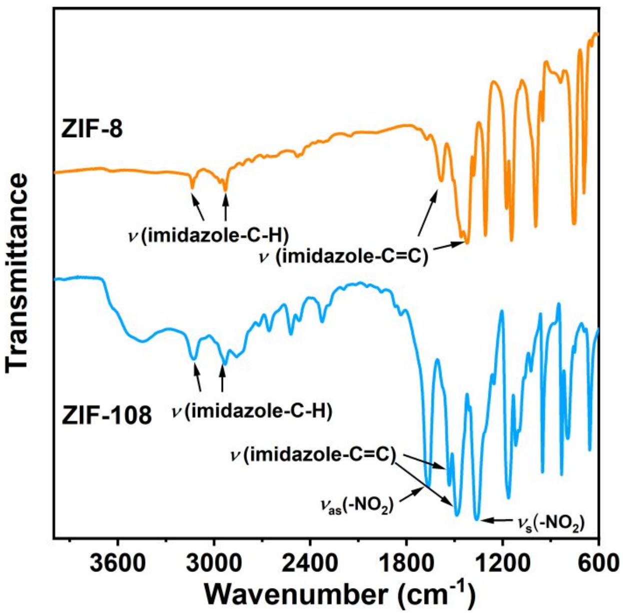

Figure S3. FT-IR characterization of ZIF-8 and ZIF-108.

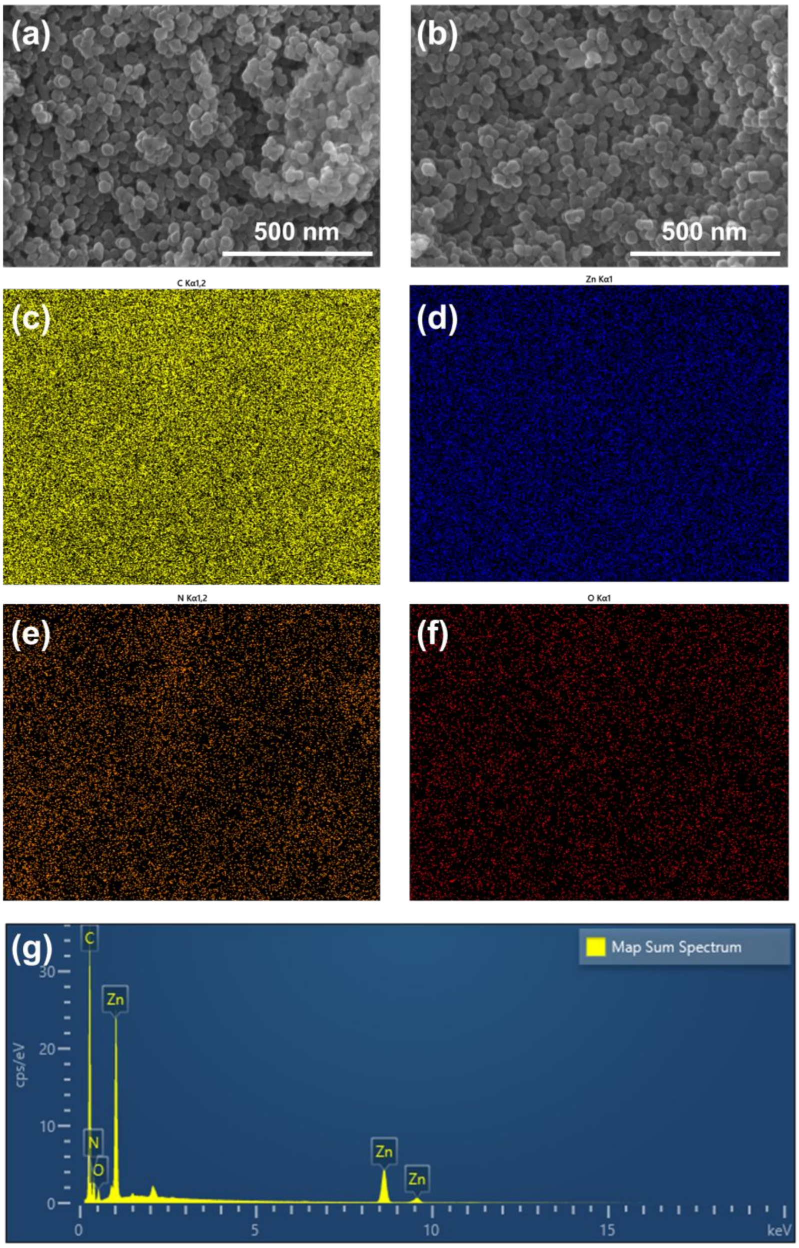

Figure S4. The SEM images of ZIF-8 as-synthesized (a) and activated ZIF-8 (b); The EDS maps of activated ZIF-8 of C (c), Zn (d), N (e) and O (f) corresponding to (b); The map sum spectrum of activated ZIF-8 corresponding to (b).

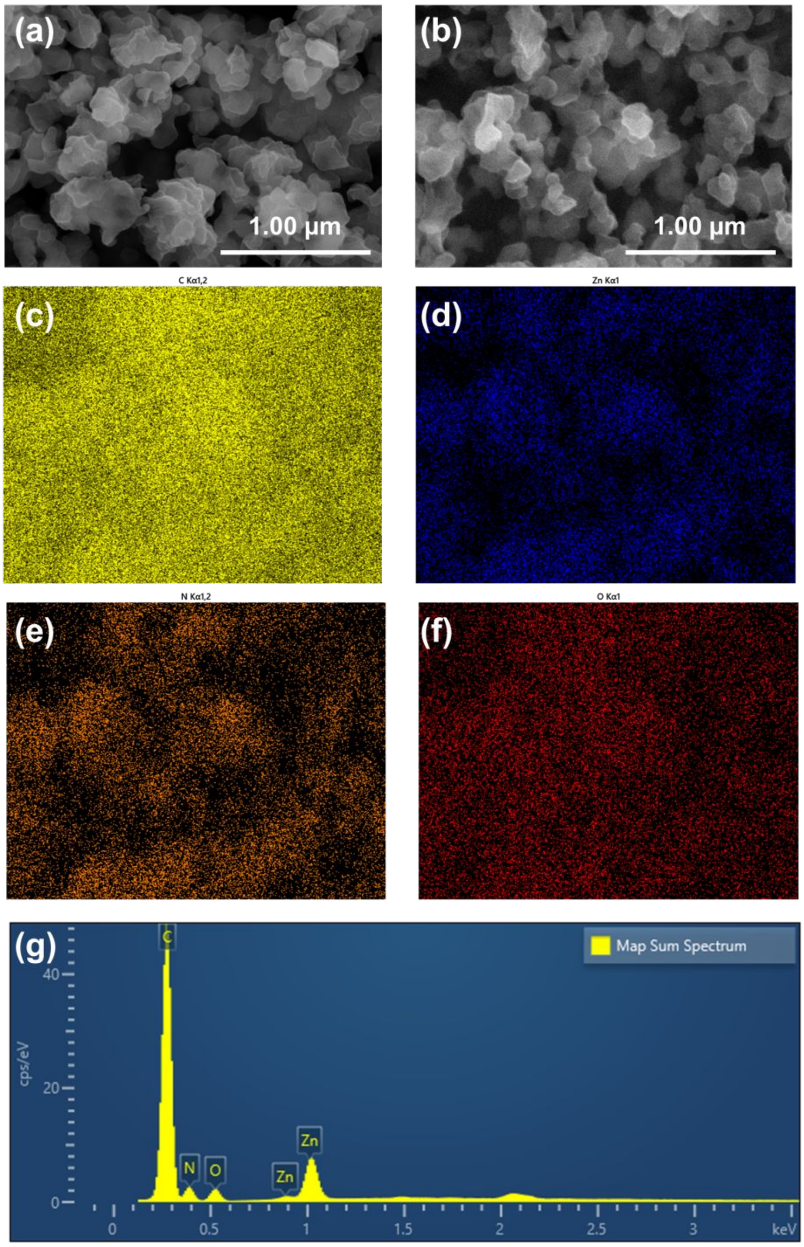

Figure S5. The SEM images of ZIF-108 as-synthesized (a) and activated ZIF-108 (b); The EDS maps of activated ZIF-108 of C (c), Zn (d), N (e) and O (f) corresponding to (b); The map sum spectrum of activated ZIF-8 corresponding to (b).

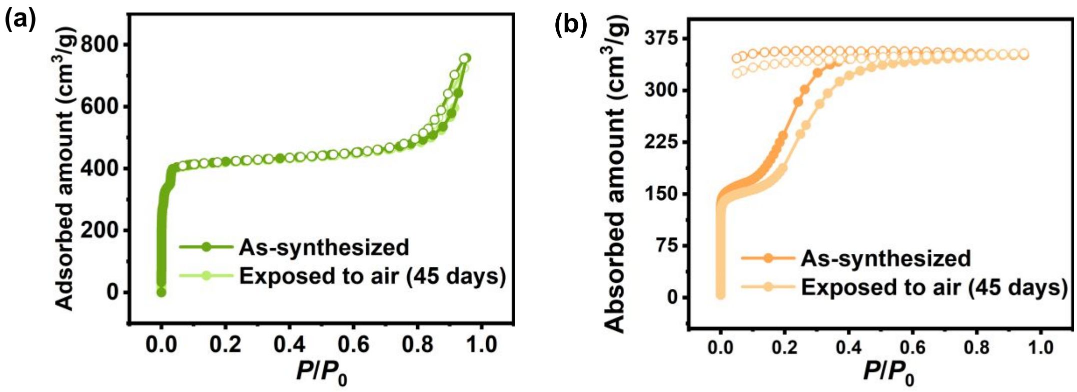

Figure S6. The N_2 adsorption isotherms in 77 K on ZIF-8 (a) and ZIF-108 (b).

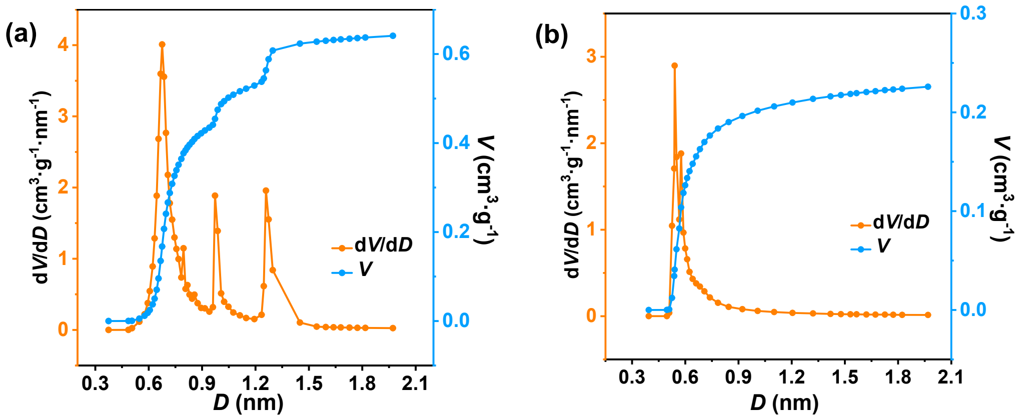

Figure S7. Pore size distributions calculated by Horvath—Kawazoe model of (a) ZIF-8 and (b) ZIF-108.

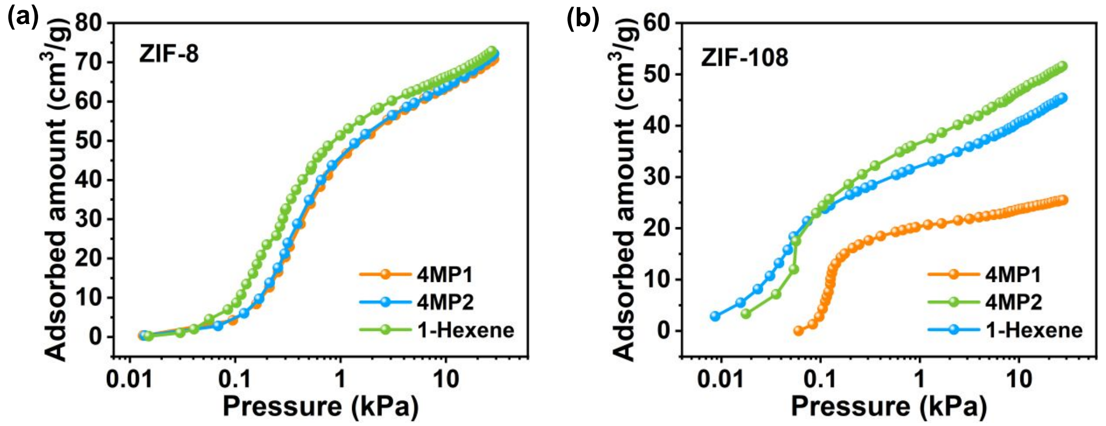

Figure S8. The adsorption isotherms of 4MP1, 4MP2, and 1-Hex on ZIF-8 (a) and ZIF-108 (b) at 333 K.

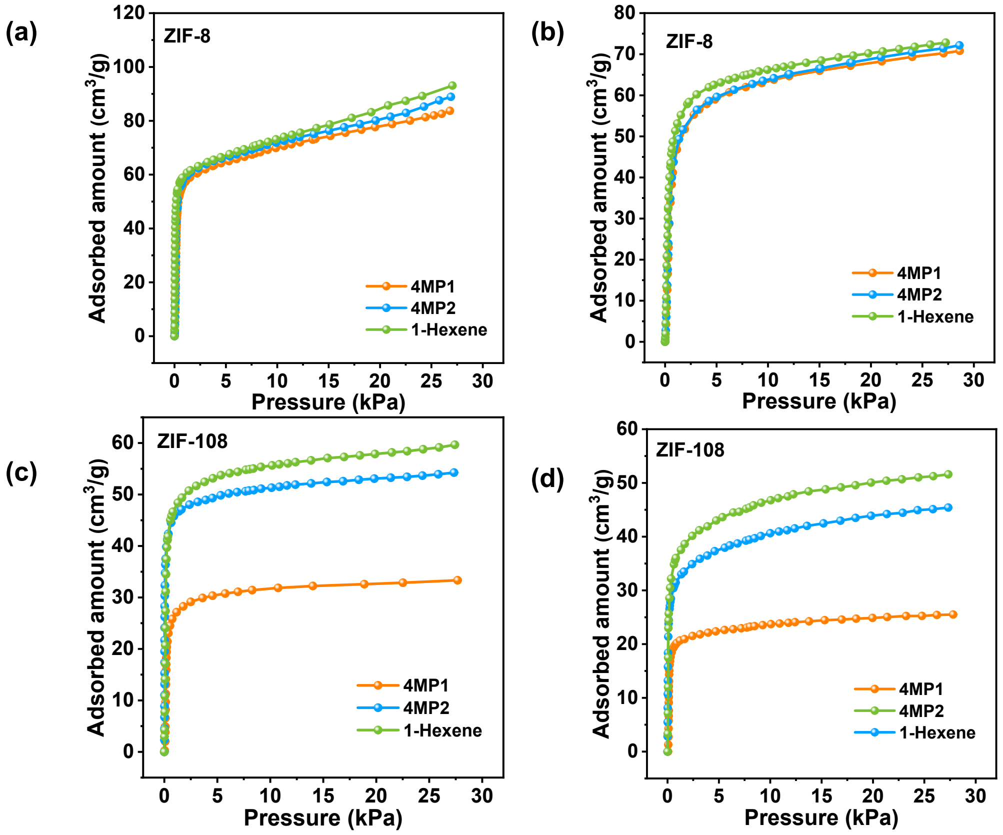

Figure S9. The linear adsorption isotherms of 4MP1, 4MP2, and 1-Hex on ZIF-8 at 303 K (a) and 333 K (b); The linear adsorption isotherms of 4MP1, 4MP2, and 1-Hex on ZIF-108 at 303 K (c) and 333 K (d).

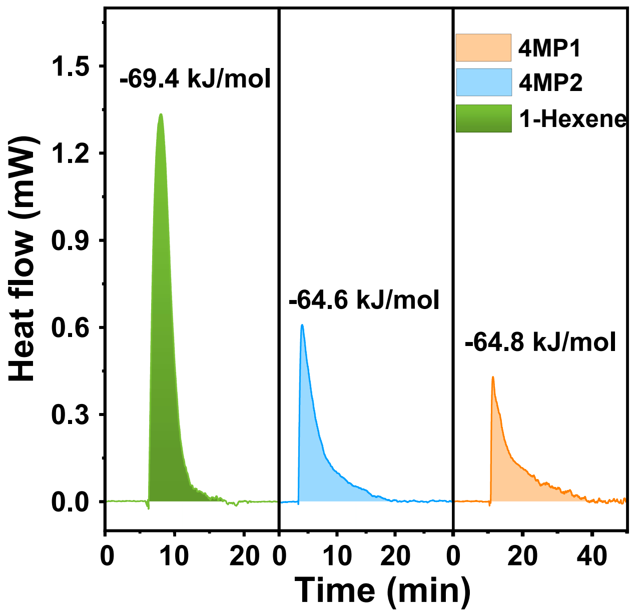

Figure S10. The adsorption heat of ZIF-8 for 4MP1, 4MP2, and 1-Hexene determined by DSC.

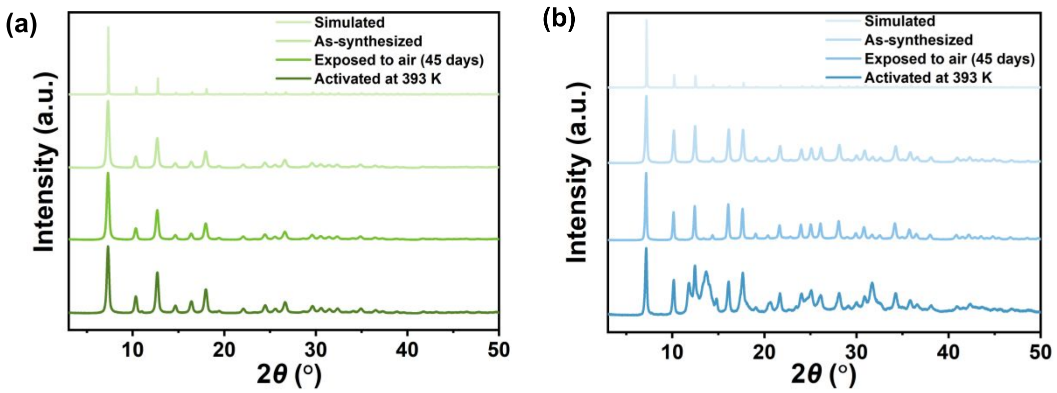

Figure S11. PXRD patterns of ZIF-8 (a) and ZIF-108 (b).

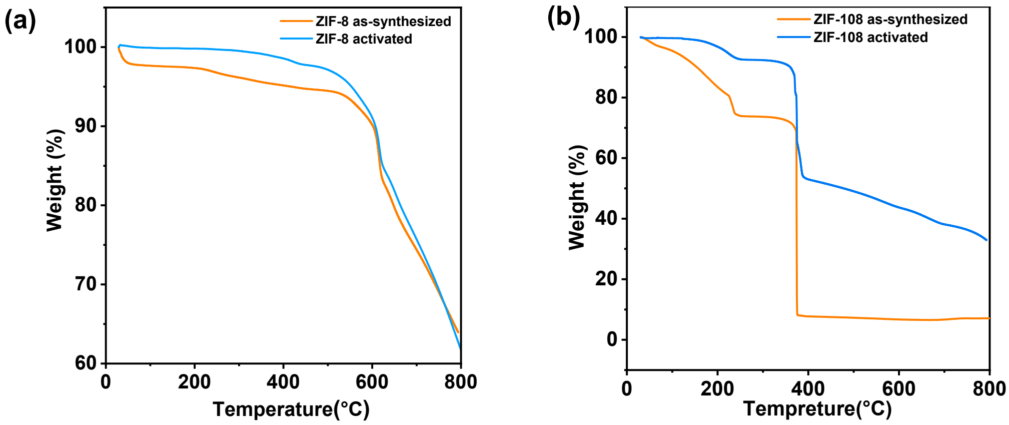

Figure S12. The TGA curves of ZIF-8 (a) and ZIF-108 (b) under N_2 atmosphere.

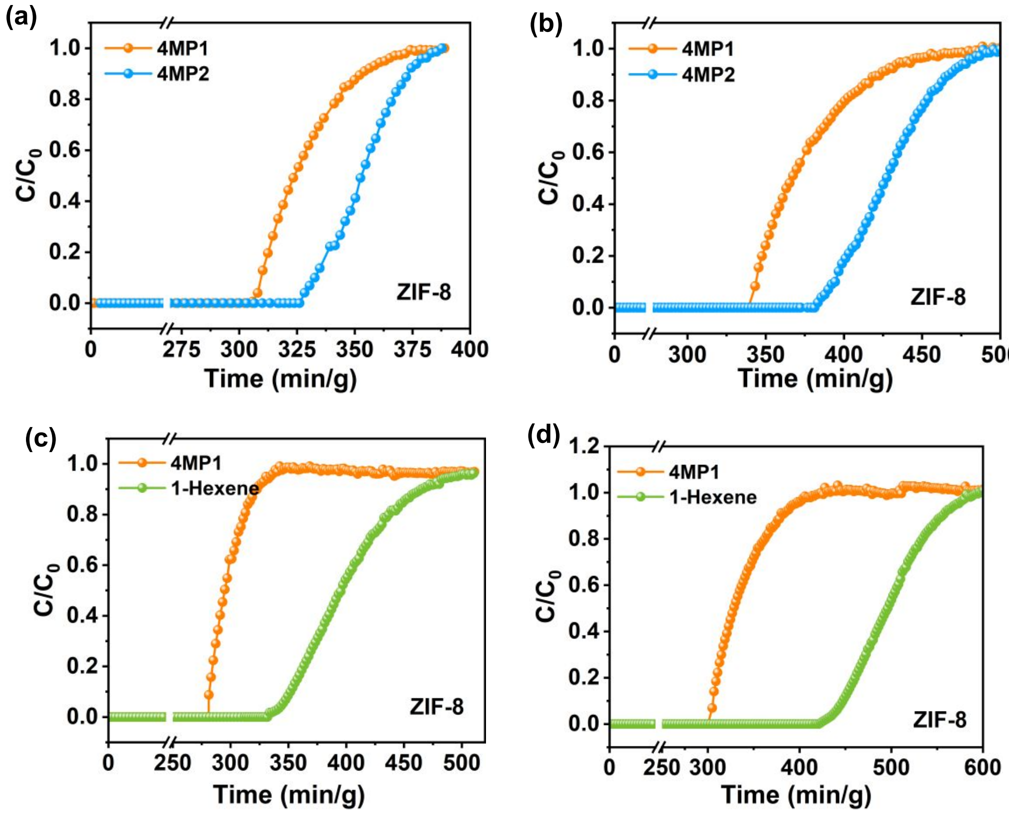

Figure S13. The fixed-bed breakthrough curves of 4MP1/4MP2 (18/1) on ZIF-8 at 303 K (a) and 333 K (b); The fixed-bed breakthrough curves of 4MP1/1-Hex (18/1) on ZIF-108 at 303 K (c) and 333 K (d).

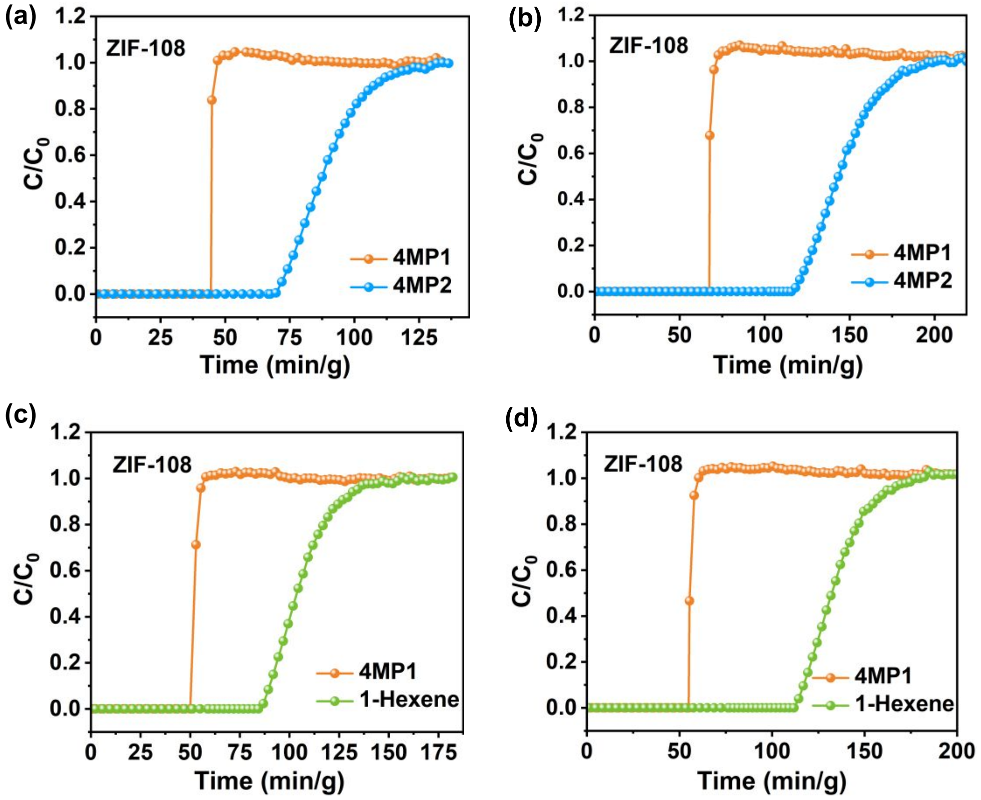

Figure S14. The fixed-bed breakthrough curves of 4MP1/4MP2 (18/1) on ZIF-108 at 303 K (a) and 333 K (b); The fixed-bed breakthrough curves of 4MP1/1-Hex (18/1) on ZIF-108 at 303 K (c) and 333 K (d).

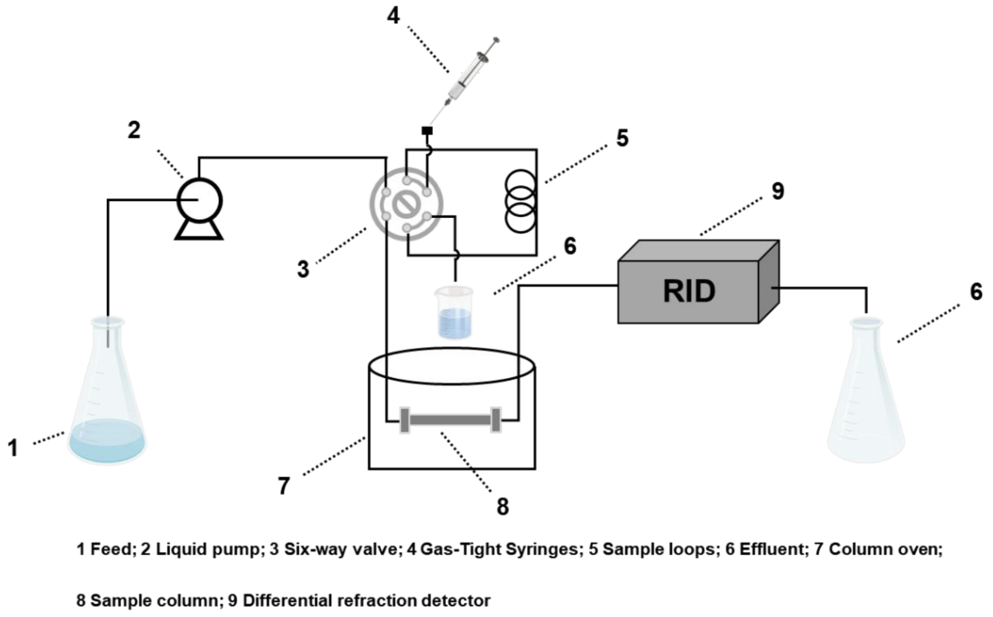

Figure S15. The scheme of pulse elution profiles measurement.

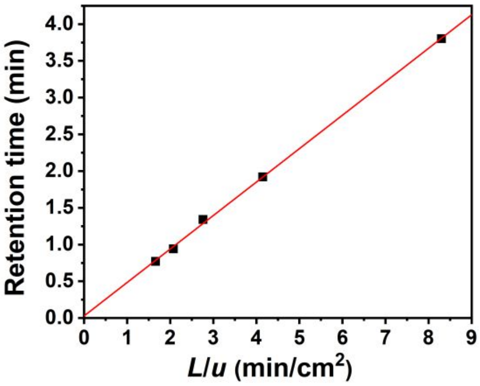

Figure S16. The relationship between the retention time and L/u on ZIF-108 column. u is the superficial velocity of mobile phase; L is length of the column.

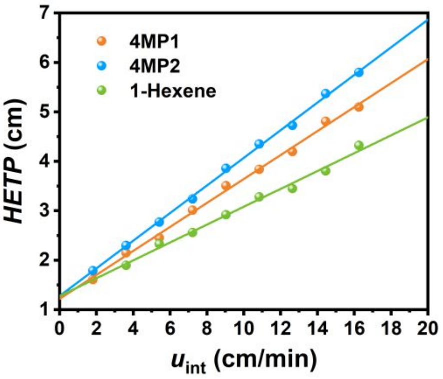

Figure S17. *HETP* of 4MP1, 4MP2, and 1-Hex of ZIF-108 column vs. the interstitial velocity of mobile phase. *HETP* is the height equivalent to a theoretical plate.

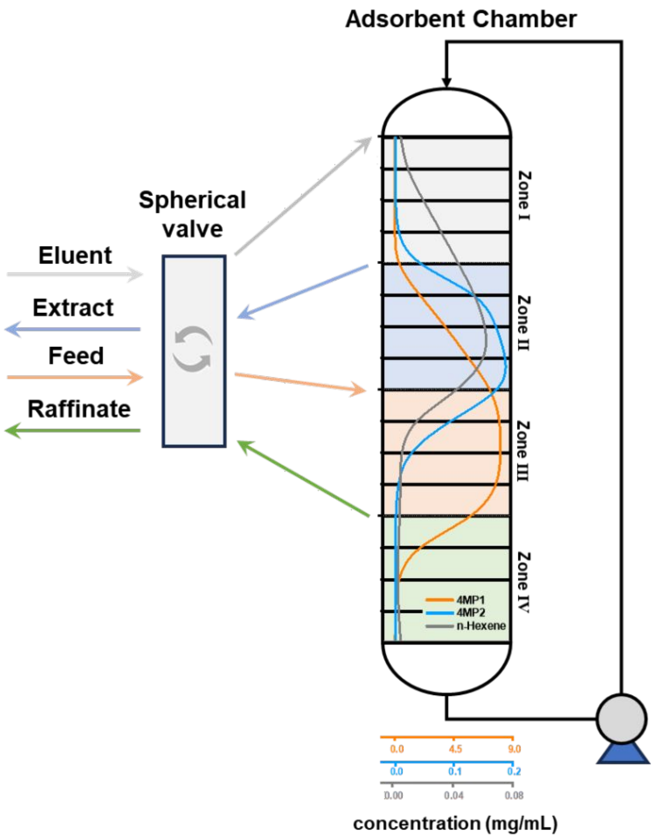

Figure S18. The scheme of SMB process and the concentration profiles in ZIF-108 column.

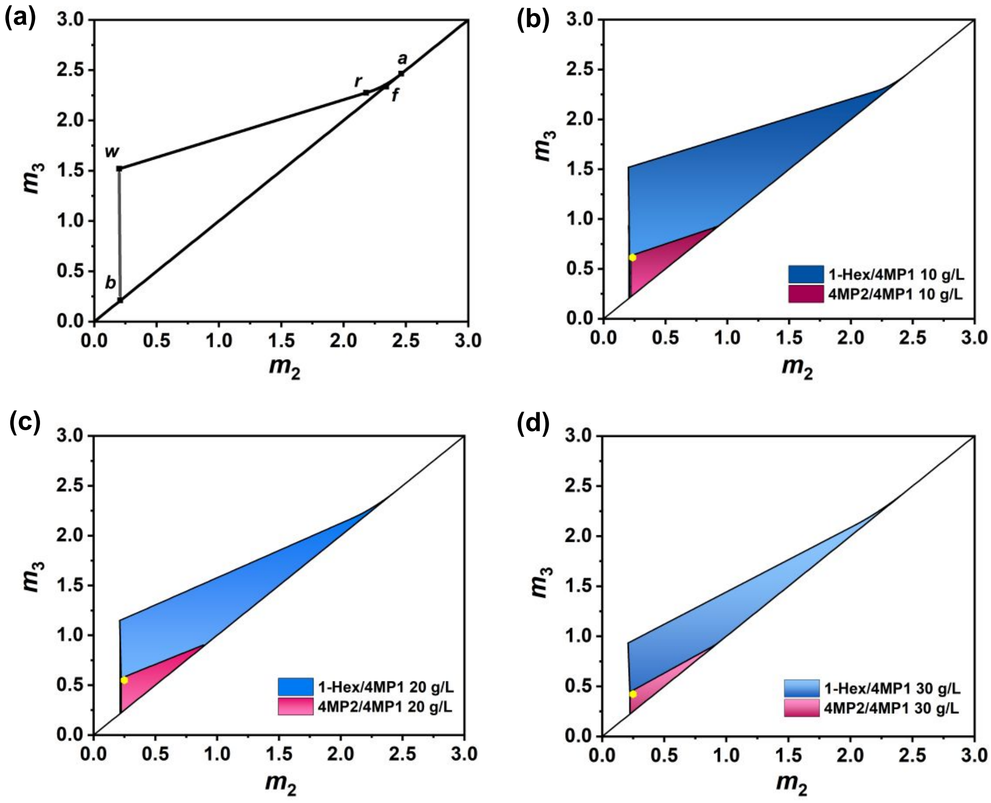

Figure S19. Schematic diagram of the calculation of the complete separation interval (a); Complete separation intervals in feeds with concentrations of 10 g/L (b), 20 g/L (c), and 30 g/L (d) on ZIF-108. m_2 and m_3 are dimensionless flow rates in zone Ⅱ and zone Ⅲ, respectively; The yellow point is chosen as operating point.

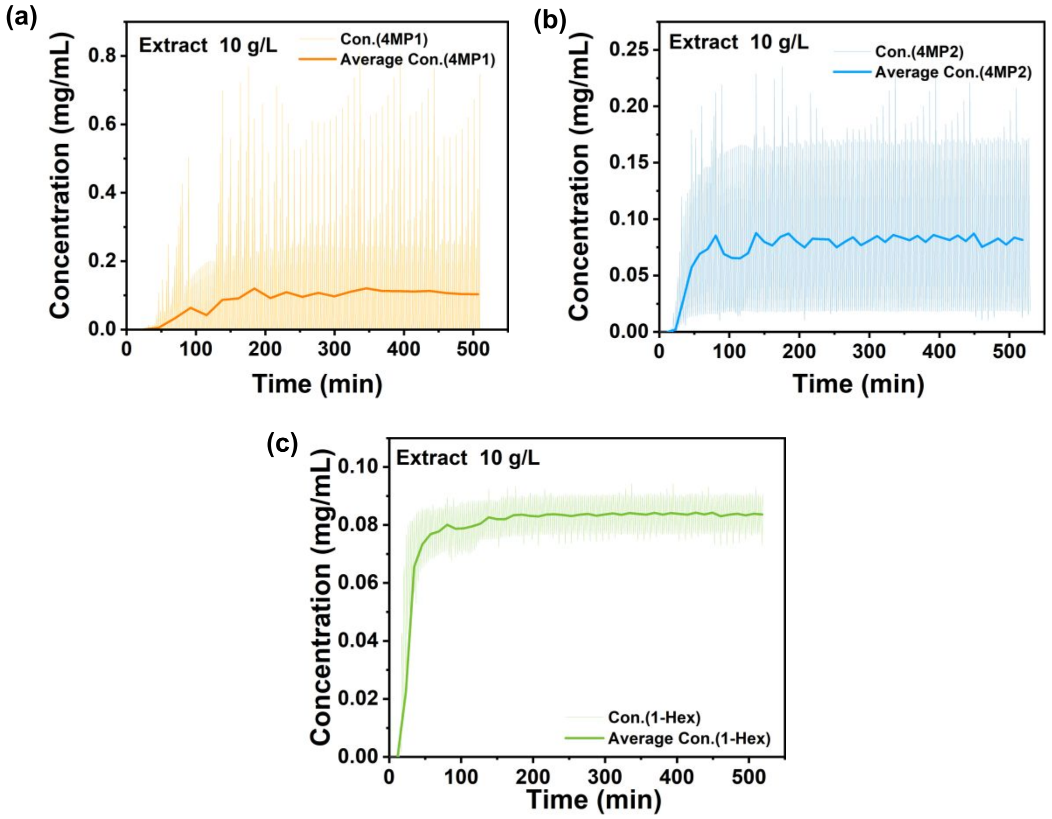

Figure S20. Concentration profiles of 4MP1 (a), 4MP2 (b), and 1-Hexene (c) in the extract from a 10 g/L feed on a ZIF-108 column during the SMB simulation process.

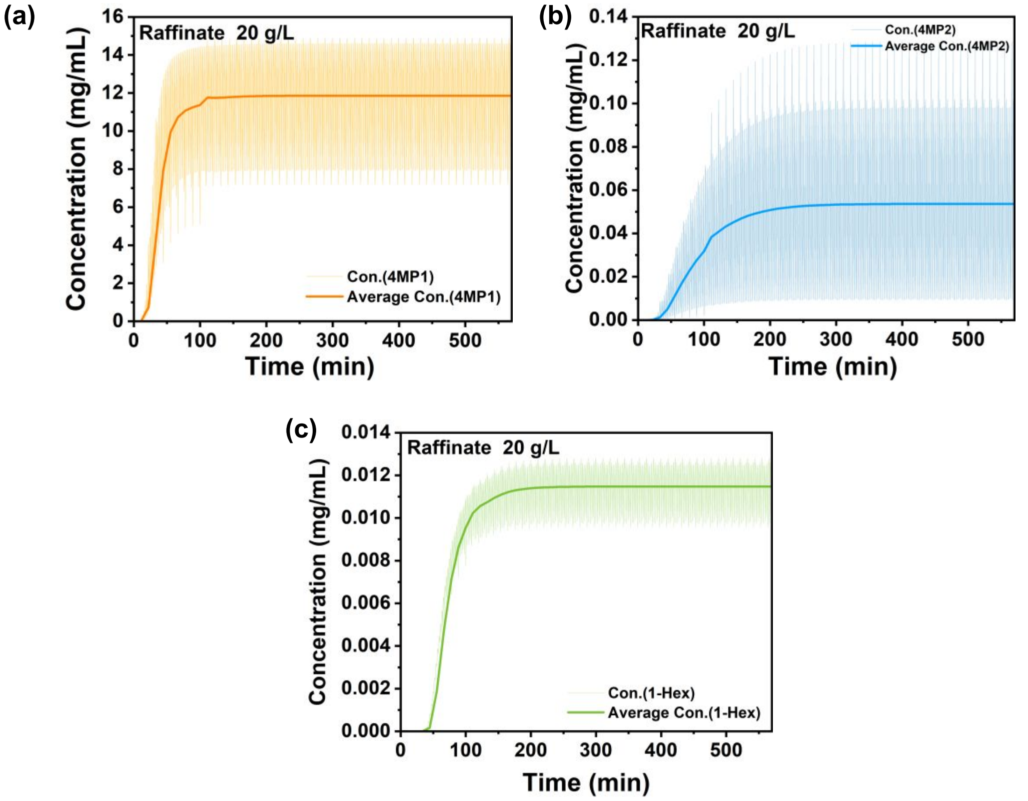

Figure S21. The concentration of 4MP1 (a), 4MP2 (b), and 1-Hexene (c) in the raffinate from a 20 g/L feed on ZIF-108 column in SMB simulation process.

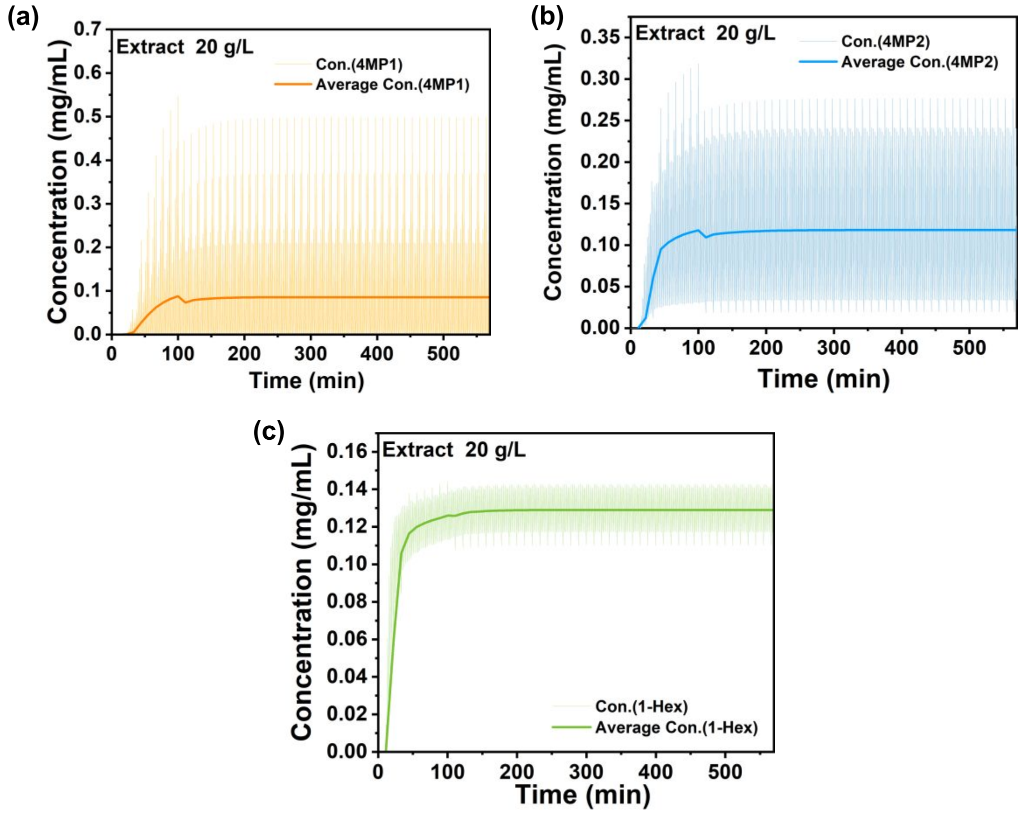

Figure S22. The concentration of 4MP1 (a), 4MP2 (b), and 1-Hexene (c) in the extract from a 20 g/L feed on ZIF-108 column in SMB simulation process.

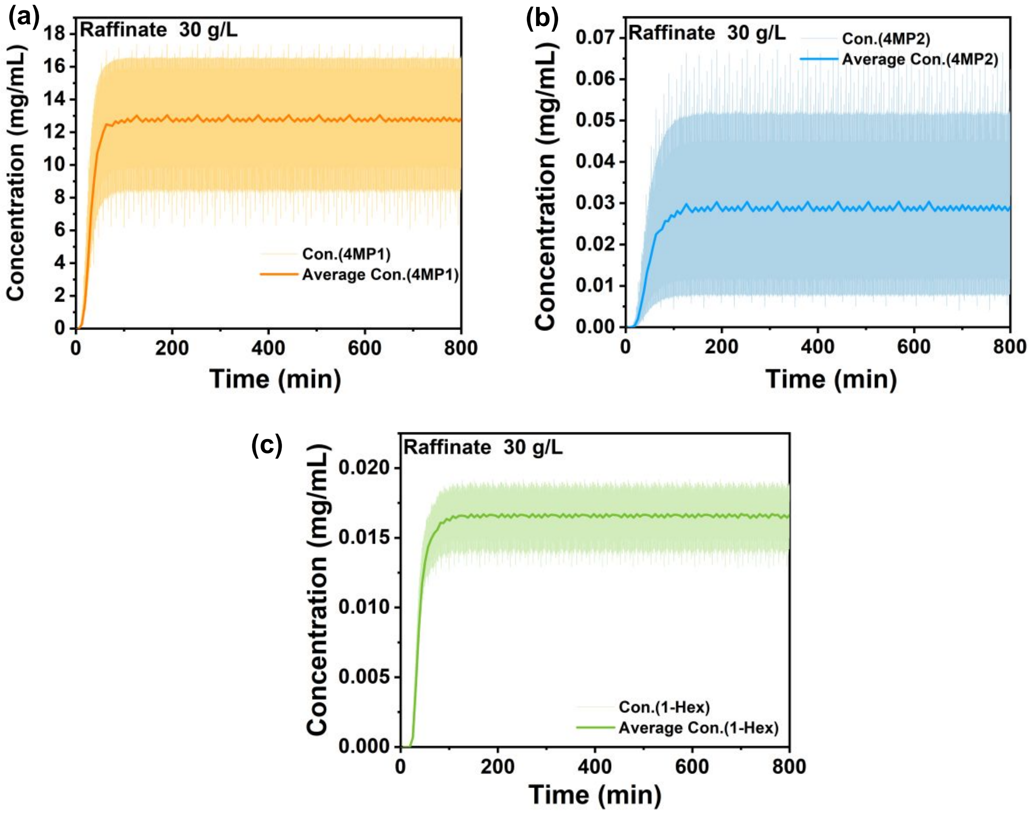

Figure S23. The concentration of 4MP1 (a), 4MP2 (b), and 1-Hexene (c) in the raffinate from a 30 g/L feed on ZIF-108 column in SMB simulation process.

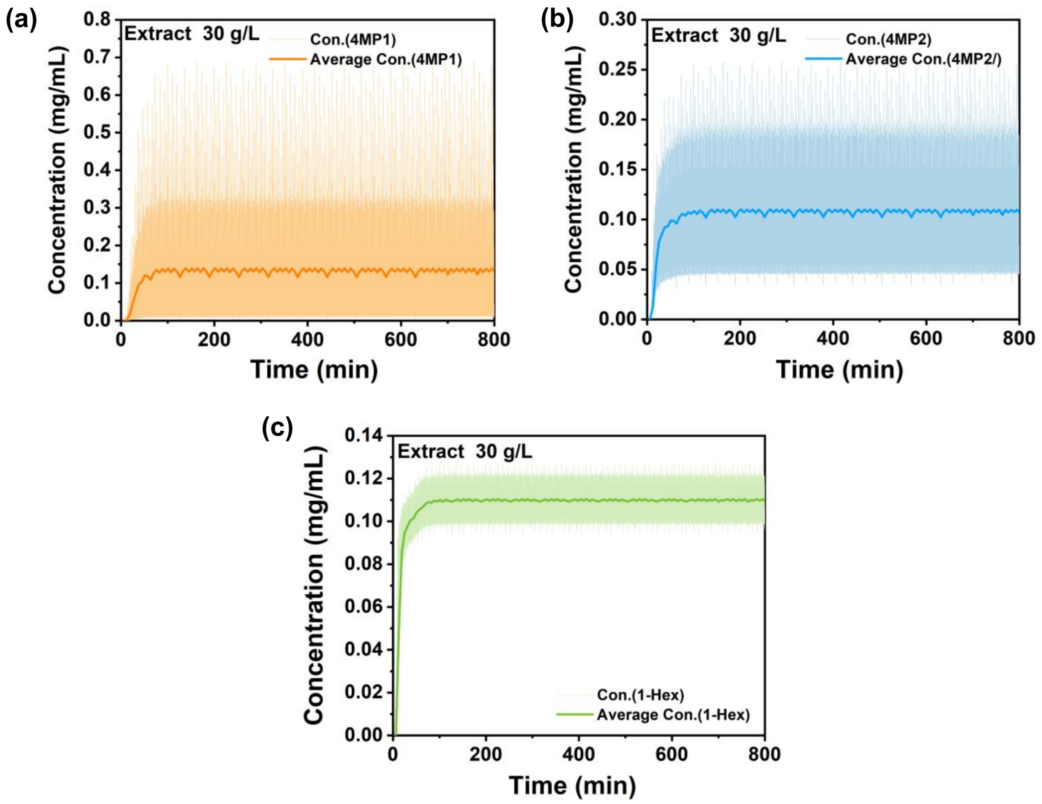

Figure S24. The concentration of 4MP1 (a), 4MP2 (b), and 1-Hexene (c) in the extract from a 30 g/L feed on ZIF-108 column in SMB simulation process.

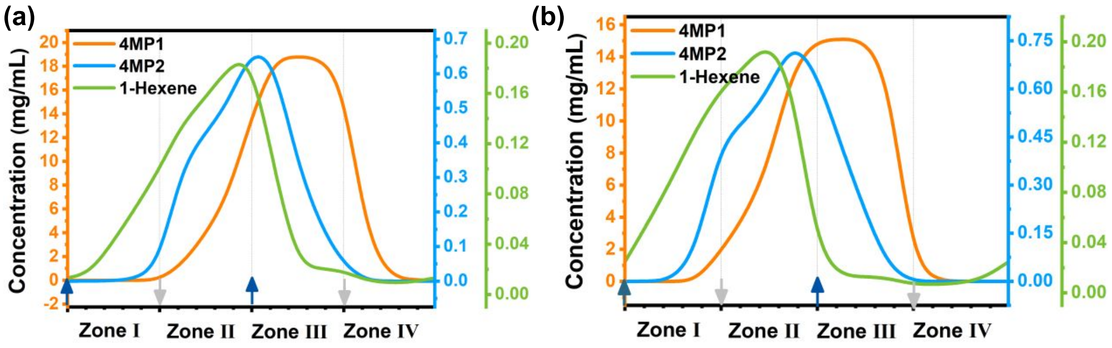

Figure S25. Internal profiles obtained at the middle of the switching time from the feed of 20 g/L (a) and 30 g/L (b) at cyclic steady state of ZIF-108. The grey downward arrow represents the outflow stream; The turquoise upward arrow represents the feed stream strand.

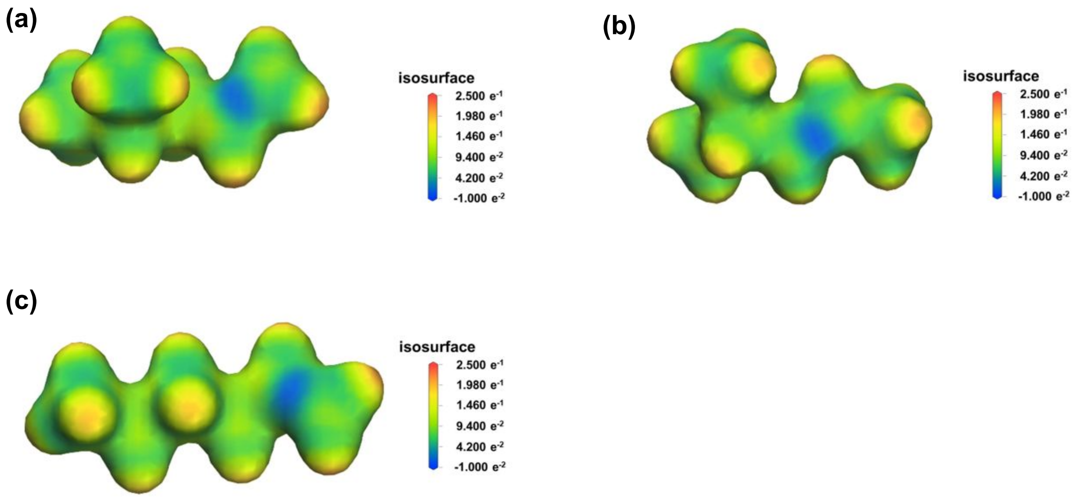

Figure S26. The surface electrostatic potential of 4MP1 (a), 4MP2 (b) and 1-Hex (c) mapped onto the 0.02 Hartree/e density isosurface with a scale spanning from -0.01 Hartree/e (blue) through 0 to 0.25 Hartree/e (red).

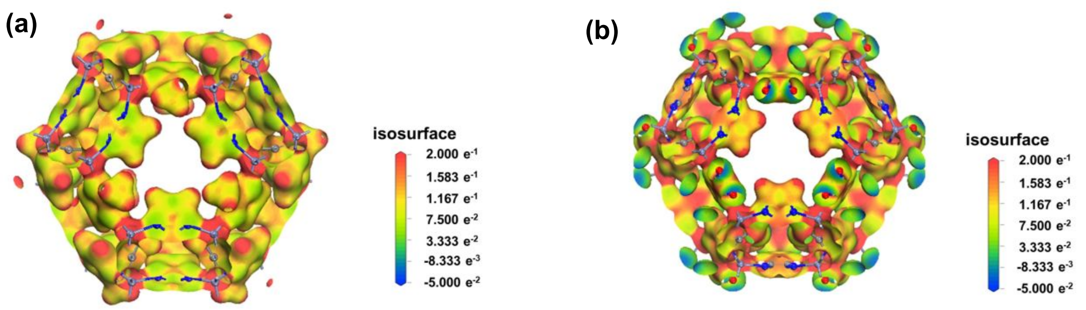

Figure S27. The surface electrostatic potential of ZIF-8 (a) and ZIF-108 (b) mapped onto the 0.02 Hartree/e density isosurface with a scale spanning from -0.05 Hartree/e (blue) through 0 to 0.20 Hartree/e (red).

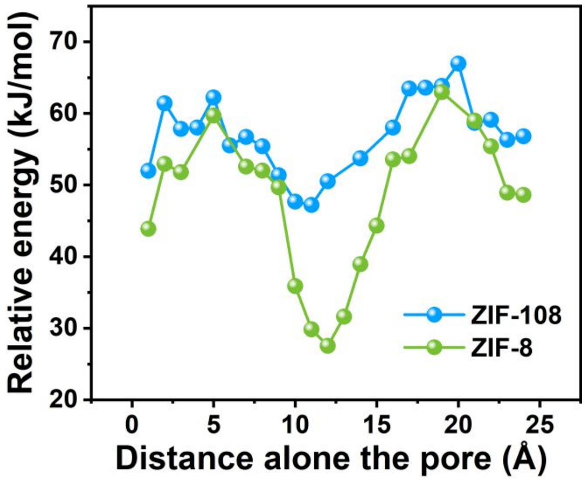

Figure S28. Energy variation of 4MP1 during the diffusion pathway in the pore channel of ZIF-8 and ZIF-108.

Table S1. Crystal data and structure refinement results for ZIF-8 and ZIF-108

**Identification code**	**ZIF-8**	**ZIF-108**
Empirical formula	C8 H10 N4 Zn	C6 H4 N6 O4 Zn
Formula weight	227.57	289.51
Temperature (K)	193	193
Space group	I-43m	I-43m
Cell	a=16.9998 (3) Å<br>b=16.9998 (3) Å<br>c=16.9998 (3) Å<br>α=90°, β=90°, γ=90°	a=17.3154 (3) Å<br>b=17.3154 (3) Å<br>c =17.3154 (3) Å<br>α=90°, β=90°, γ=90°
Volume (Å^3)	4912.83 (3)	5191.56 (3)
Mu (mm^{-1})	1.24	1.41
Dc (g/cm^3)	0.90	1.09
Z	12	12
F000	1392	1728
h, k, l_{max}	22, 18, 22	27, 17, 10
N_{ref}	15583	16640
T_{min}, T_{max}	0.886, 0.855	0.935, 0.920

Table S2. BET surface areas and pore volumes of samples

Sample	As synthesized		Exposed to air for 45 days	
	S_{BET} (m^2/g)	V_{total} (cm^3/g)	S_{BET} (m^2/g)	V_{total} (cm^3/g)
ZIF-8	1454.30	0.67	1374.81	0.56
ZIF-108	945.36	0.62	755.81	0.52

Table S3. Summary of vapor uptake of 4MP1, 4MP2, and 1-Hexene, and uptake ratios of 4MP2/4MP1 and 1-Hex/4MP1

**Materials**	Temperature (K)	4MP1 uptake (cm^3/g)	4MP2 uptake (cm^3/g)	1-Hex uptake (cm^3/g)	Uptake ratio of 4MP2/4MP1	Uptake ratio of 1-Hex/4MP1	Synthesis	Purchase[a]
13X	303	36.57	36.00	30.93	0.97	0.83		Sigma Aldrich
ZSM-5	303	32.42	2.44	29.32	0.10	1.13		XFNANO
4A-Na	303	2.91	2.34	3.68	0.79	1.21		XFNANO
CAU-10	303	34.76	44.81	56.42	1.31	1.41	Reinsch *et al* [10]	
Cu-BTC	303	80.51	82.32	85.41	1.02	1.05	Chui *et al* [11]	
MIL-53 (Al)	303	69.4	81.13	86.13	1.16	1.16	Loiseau *et al* [12]	
UIO-66	303	51.12	57.53	69.31	1.12	1.22	Cavka *et al* [13]	
NU-2200	303	27.94	34.22	37.22	1.22	1.33	Lal *et al* [14]	
ZIF-8	303	82.57	87.58	92.32	1.06	1.11	This work	
ZIF-108	303	33.10	54.25	59.67	1.64	1.80	This work	

[a] Used without further purification

Table S4. The parameters of kinetic diffusion at 303 K

**Parameters**	ZIF-8			ZIF-108		
	4MP1	4MP2	1-Hexene	4MP1	4MP2	1-Hexene
D_S/r^2 (10^{-5} s^{-1})	1.42	6.63	516.32	203	190	1213
R^2	0.96	0.97	0.98	0.97	0.98	098

Table S5. Separation performance of 4MP1, 4MP2, and 1-Hex mixtures on ZIF-8 at 303 K

		4MP1/4MP2/1-Hex (18/1/1)	4MP1/4MP2 (18/1)	4MP1/1-Hex (18/1)
Dynamic Adsorption (mmol/g)	4MP1	2.81	4.01	2.80
	4MP2	0.16	0.26	--
	1-Hex	0.26	--	0.26
Breakthrough time (min)	4MP1	318.12	306.37	300.40
	4MP2	356.13	342.68	--
	1-Hex	461.32	--	419.20
Selectivity	4MP2/4MP1	1.02	1.15	--
	1-Hex/4MP1	1.66	--	1.70
Productivity (>99.9%, mmol/g)	4MP1	0.168	0.180	0.740

Table S6. Separation performance of 4MP1, 4MP2, and 1-Hex mixtures on ZIF-8 at 333 K

		4MP1/4MP2/1-Hex (18/1/1)	4MP1/4MP2 (18/1)	4MP1/1-Hex (18/1)
Dynamic Adsorption (mmol/g)	4MP1	2.51	3.60	2.44
	4MP2	0.14	0.22	--
	1-Hex	0.21	--	0.21
Breakthrough time (min)	4MP1	290.56	274.86	280.25
	4MP2	309.68	293.42	--
	1-Hex	377.25	--	329.55
Selectivity	4MP2/4MP1	1.01	1.07	--
	1-Hex/4MP1	1.54	--	1.51
Productivity (>99.9%, mmol/g)	4MP1	0.061	0.065	0.456

Table S7. Separation performance of 4MP1, 4MP2, and 1-Hex mixtures on ZIF-108 at 303 K

		4MP1/4MP2/1-Hex (18/1/1)	4MP1/4MP2 (18/1)	4MP1/1-Hex (18/1)
Dynamic Adsorption (mmol/g)	4MP1	0.720	0.550	0.471
	4MP2	0.088	0.085	--
	1-Hex	0.096	--	0.090
Breakthrough time (min)	4MP1	51.0	53.6	43.5
	4MP2	81.3	93.4	--
	1-Hex	96.6	--	89.3
Selectivity	4MP2/4MP1	2.2	2.9	--
	1-Hex/4MP1	2.4	--	3.4
Productivity (>99.9%, mmol/g)	4MP1	0.458	0.456	0.779

Table S8. Separation performance of 4MP1, 4MP2, and 1-Hex mixtures on ZIF-108 at 333 K

		4MP1/4MP2/1-Hex (18/1/1)	4MP1/4MP2 (18/1)	4MP1/1-Hex (18/1)
Dynamic Adsorption (mmol/g)	4MP1	0.523	0.507	0.443
	4MP2	0.056	0.069	--
	1-Hex	0.068	--	0.074
Breakthrough time (min)	4MP1	44.07	40.04	43.5
	4MP2	57.88	62.92	--
	1-Hex	69.11	--	89.3
Selectivity	4MP2/4MP1	1.9	2.3	--
	1-Hex/4MP1	2.3	--	2.1
Productivity (>99.9%, mmol/g)	4MP1	0.241	0.374	0.431

Table S9. Design and model parameters for SMB

**Parameters**	
ZIF-108 column length (mm)	250
ZIF-108 column diameter (mm)	4.6
Total porosity (ε_t)	0.45
Bed porosity (ε)	0.30
Internal porosity (ε_p)	0.21
H_{4MP1}	0.23
H_{4MP2}	0.95
$H_{1\text{-Hex}}$	2.47
b_{4MP1} (mL/mg)	0.06
b_{4MP2} (mL/mg)	0.06
$b_{1\text{-Hex}}$ (mL/mg)	0.19
D_{ax} (cm^2/min)	$0.62u_{int}$
MTC_{4MP1} (min^{-1})	1.62
MTC_{4MP2} (min^{-1})	2.93
$MTC_{1\text{-Hex}}$ (min^{-1})	6.62

H_i is Henry constant; b_i is equilibrium constant; D_{ax} is the axial dispersion coefficient; *MTCs* are the mass transfer coefficients.

Table S10. The parameters and selectivity of liquid chromatographic column of ZIF-108

	H	Q (mg/mL)	b	k
4MP1	0.23	3.83	0.06	/
4MP2	0.95	15.01	0.06	4.13
1-Hex	2.47	13.02	0.19	10.74

H_i is Henry constant; b_i is equilibrium constant; Q is the most adsorption amount; k is Herry selectivity.

Table S11. The operating parameters of SMB using the feed with different concentration

Operating Parameter	10 g/L	20 g/L	30 g/L
Switch time (min)	2.88	2.22	1.26
m_1 (mL/min)	2.47	0.25	2.47
m_2 (mL/min)	0.24	0.25	0.26
m_3 (mL/min)	0.61	0.55	0.42
m_4 (mL/min)	0.15	0.12	0.10
Feed flow (mL/min)	0.30	0.30	0.30
Eluent flow (mL/min)	1.83	4.29	4.29
Raffinate flow (mL/min)	0.37	0.59	0.59
Extract flow (mL/min)	1.8	2.28	4.00
Ⅰ zone (mL/min)	2.60	3.38	5.95
Ⅱ zone (mL/min)	0.83	1.10	1.94
Ⅲ zone (mL/min)	1.13	1.40	2.25
Ⅳ zone (mL/min)	0.767	0.96	1.65

Table S12. The performance of SMB of ZIF-108 at 303 K

	10 g/L	20 g/L	30 g/L
$Purity_{4MP1}$	99.8%	99.5%	99.6%
C_{4MP1}(g/L)	7.12	11.88	12.8
C_{4MP2}(g/L)	0.007	0.053	0.028
$C_{1\text{-}Hexene}$(g/L)	0.005	0.011	0.0165
Recovery rate of 4MP1	96.5%	97.4%	93.7%

Authors contributions

Zongbi Bao conceived and designed the study. Zhe Chu performed the experiments and created the pictures; Fuqiang Chen wrote the original manuscript and assisted with figure preparation and data analysis; Jiaqi Li, Yifeng Cao, Lihang Chen, Feng Zhou, Huixia Ma participated in the discussion. Zhiguo Zhang, Qiwei Yang, Kai Qiao, Qilong Ren, and Zongbi Bao reviewed and edited the manuscript. Zongbi Bao acquired funding and supervised the project. All authors read and approved the final manuscript.

References

1. Chang, N.; Gu, Z. Y.; Yan, X. P., Zeolitic Imidazolate Framework-8 Nanocrystal Coated Capillary for Molecular Sieving of Branched Alkanes from Linear Alkanes along with High-Resolution Chromatographic Separation of Linear Alkanes. *J. Am. Chem. Soc.* **2010**, *132* (39), 13645-13647.
2. Ban, Y. J.; Li, Y. S.; Peng, Y.; Jin, H.; Jiao, W. M.; Liu, X. L.; Yang, W. S., Metal-Substituted Zeolitic Imidazolate Framework ZIF-108: Gas-Sorption and Membrane-Separation Properties. *Chem.-Eur. J.* **2014**, *20* (36), 11402-11409.
3. Bao, Z. B.; Su, B. G.; Ren, Q. L., Kinetic and equilibrium study of the enantioseparation of paroxetine intermediate on amylose and tartaric acid-based chiral stationary phases. *J. Sep. Sci.* **2008**, *31* (1), 16-22.
4. Wang, X.; Liu, Y.; Ching, C. B., Kinetic and equilibrium study of enantioseparation of propranolol in preparative scale chromatography. *Sep. Purif. Technol.* **2006**, *50* (2), 204-211.
5. Zabka, M.; Minceva, M.; Gornes, P. S.; Rodrigues, A. E., Chiral separation of R,S-α-tetralol by Simulated Moving Bed. *Sep. Sci. Technol.* **2008**, *43* (4), 727-765.
6. Rajendran, A.; Paredes, G.; Mazzotti, M., Simulated moving bed chromatography for the separation of enantiomers. *J. Chromatogr. A* **2009**, *1216* (4), 709-738.
7. Seidel-Morgenstern, A., Experimental determination of single solute and competitive adsorption isotherms. *J. Chromatogr. A* **2004**, *1037* (1-2), 255-272.
8. Huang, Y.; Wan, J.; Pan, T.; Ge, K.; Guo, Y.; Duan, J.; Bai, J.; Jin, W.; Kitagawa, S., Delicate Softness in a Temperature-Responsive Porous Crystal for Accelerated Sieving of Propylene/Propane. *J. Am. Chem. Soc.* **2023**, *145* (44), 24425-24432.
9. Jiang, Z.-J.; Wang, Y.; Luo, D.; Wei, R.-J.; Lu, W.; Li, D., Dehydration-Induced Cluster Consolidation in a Metal-Organic Framework for Sieving Hexane Isomers. *Angew. Chem. Int. Ed.* **2024**, e202403209.
10. Reinsch, H.; van der Veen, M. A.; Gil, B.; Marszalek, B.; Verbiest, T.; de Vos, D.; Stock, N., Structures, Sorption Characteristics, and Nonlinear Optical Properties of a New Series of Highly Stable Aluminum MOFs. *Chem. Mater.* **2013**, *25* (1), 17-26.
11. Chui, S. S. Y.; Lo, S. M. F.; Charmant, J. P. H.; Orpen, A. G.; Williams, I. D., A chemically functionalizable nanoporous material $[Cu_3(TMA)_2(H_2O)_3]_n$. *Science* **1999**, *283* (5405), 1148-1150.
12. Loiseau, T.; Serre, C.; Huguenard, C.; Fink, G.; Taulelle, F.; Henry, M.; Bataille, T.; Férey, G., A rationale for the large breathing of the porous aluminum terephthalate (MIL-53) upon hydration. *Chem.-Eur. J.* **2004**, *10* (6), 1373-1382.
13. Cavka, J. H.; Jakobsen, S.; Olsbye, U.; Guillou, N.; Lamberti, C.; Bordiga, S.; Lillerud, K. P., A new zirconium inorganic building brick forming metal organic frameworks with exceptional stability. *J. Am. Chem. Soc.* **2008**, *130* (42), 13850-13851.
14. Lal, B.; Idrees, K. B.; Xie, H.; Smoljan, C. S.; Shafaie, S.; Islamoglu, T.; Farha, O. K., Pore Aperture Control Toward Size-Exclusion-Based Hydrocarbon Separations. *Angew. Chem. Int. Ed.* **2023**, *62* (16), e202219053.
